# Incorporating Stage-Specific Drug Action into Pharmacological Modeling of Antimalarial Drug Treatment

**DOI:** 10.1128/AAC.01172-15

**Published:** 2016-04-22

**Authors:** Eva Maria Hodel, Katherine Kay, Ian M. Hastings

**Affiliations:** Liverpool School of Tropical Medicine, Liverpool, United Kingdom

## Abstract

Pharmacological modeling of antiparasitic treatment based on a drug's pharmacokinetic and pharmacodynamic properties plays an increasingly important role in identifying optimal drug dosing regimens and predicting their potential impact on control and elimination programs. Conventional modeling of treatment relies on methods that do not distinguish between parasites at different developmental stages. This is problematic for malaria parasites, as their sensitivity to drugs varies substantially during their 48-h developmental cycle. We investigated four drug types (short or long half-lives with or without stage-specific killing) to quantify the accuracy of the standard methodology. The treatment dynamics of three drug types were well characterized with standard modeling. The exception were short-half-life drugs with stage-specific killing (i.e., artemisinins) because, depending on time of treatment, parasites might be in highly drug-sensitive stages or in much less sensitive stages. We describe how to bring such drugs into pharmacological modeling by including additional variation into the drug's maximal killing rate. Finally, we show that artemisinin kill rates may have been substantially overestimated in previous modeling studies because (i) the parasite reduction ratio (PRR) (generally estimated to be 10^4^) is based on observed changes in circulating parasite numbers, which generally overestimate the “true” PRR, which should include both circulating and sequestered parasites, and (ii) the third dose of artemisinin at 48 h targets exactly those stages initially hit at time zero, so it is incorrect to extrapolate the PRR measured over 48 h to predict the impact of doses at 48 h and later.

## INTRODUCTION

Identifying optimal deployment policies and improved drug stewardship (for example, suppression of monotherapies and detection of counterfeit drugs) have become major public health objectives designed to minimize the onset of resistance to the currently recommended first-line drugs for uncomplicated malaria, i.e., artemisinin-based combination therapies (ACTs). One method to identify best practice for their deployment is pharmacological modeling of drug action. This has been widely used in other infectious diseases, notably bacteria (recently reviewed in reference 1). Its application to malaria treatment is now being strongly recommended to optimize deployment practices (2, 3), and the World Health Organization (WHO) has recommended the development of models to improve the understanding of antimalarial drug resistance and management (4). Recent examples of pharmacological modeling can be found elsewhere (5–17), although a less mechanistic approach can also be employed by fitting curves to observed clinical data (e.g., see reference 18). Pharmacological models have a potentially huge impact in contributing to the rational design and deployment of drug therapies that can potentially save several million lives annually.

**FIG 1:**
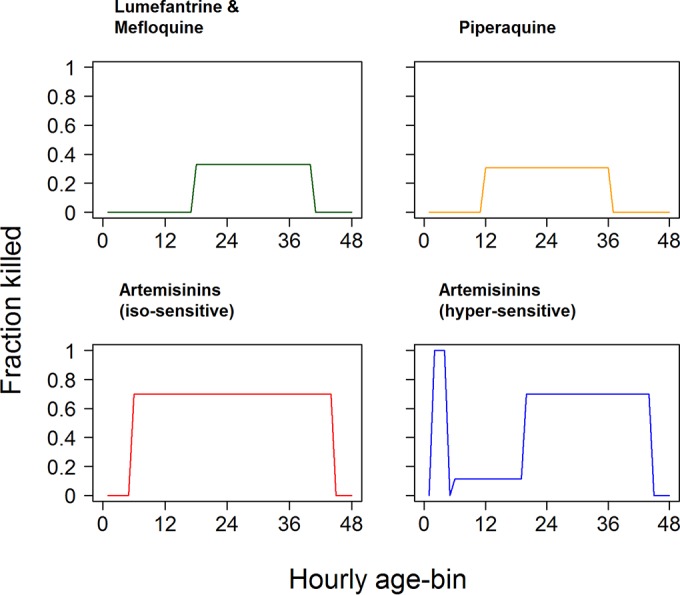
Pharmacodynamic profiles of antimalarial drugs used in the discrete-time methodology. The profiles describe the fraction of parasites killed per hour by the drug for each of the 48-h age bins (i.e., 1 − Ψ^*b*,*t*^ from equation 5). Calibrations are based on an asynchronous, “uniform” parasite infection, which results in a PRR_48_ of 10^3^ (lumefantrine, mefloquine, and piperaquine) or a PRR_48_ of 10^4^ (artemisinins). We investigated two profiles of sensitivity to artemisinins. The “isosensitive” profile assumes that all parasite stages are equally sensitive to artemisinin: this is essentially the same profile as that for partner drugs but with a wider range of stages being killed. The other “hypersensitive” profile assumes differential artemisinin killing between the stages. This seems intuitively plausible because drug sensitivity presumably depends on the metabolic processes taking place at each stage of development and also reflects recent findings that P. falciparum appears far more sensitive to artemisinins in the early ring stages than in later stages (43).

The conventional *in silico* method of predicting the therapeutic outcome of malaria treatment is to track the number of parasites following drug treatment using ordinary differential equations (ODEs) (e.g., see reference 19) (see discussion of equation 1, below). Some antimalarial drugs can act against liver stages and/or gametocytes, but it is the asexual blood stages (rings, trophozoites, schizonts, and merozoites) in human red blood cells (RBCs) that cause symptoms. In this work, we focus exclusively on modeling drug action against these asexual blood stages. This approach has one major inherent drawback when applied to malaria: it assumes that the malaria parasites within a patient are entirely homogenous, i.e., that all parasites are in identical states so that, given a certain drug concentration, all parasites are equally likely to be eliminated by the drug and, if they are not eliminated, are all equally likely to reproduce. This assumption of parasite homogeneity is violated in malaria, where a single infection may harbor individual parasites that become distinctly heterogeneous as they pass through their development processes within RBCs. Plasmodium falciparum, the most deadly of the Plasmodium species causing human malaria (20), has a characteristic 48-h infection cycle within RBCs. Parasites infect a RBC, establish several membranes and transport systems to support their subsequent development, digest and detoxify hemoglobin, and finally initiate DNA synthesis to produce 20 to 40 new parasites that emerge from the RBC when it ruptures 48 h after its infection. These developmental processes are reflected in large changes in parasite metabolism. Critically, drugs are active only against those stages that utilize metabolic processes targeted by the drugs so that drug stage specificity occurs. As an example, many partner drugs in ACTs are believed to target heme digestion/detoxification and are effective only against trophozoite and schizont stages (21), when rapid heme digestion is occurring. These partner drugs, however, have long half-lives and are present at active concentrations for several 48-h cycles after treatment, so parasites pass through all stages in the presence of the drugs, and the lack of stage specificity in the models is not conjectured to be too problematic. Partner drugs in ACTs are combined with artemisinins. Recent reports on artemisinin resistance potentially evolving in Southeast Asia led to an increased focus on their performance (22–25). It is unknown how artemisinin resistance may affect clinical impact on therapeutic outcomes, and reliance on killing effects of the partner drug in ACTs is imperative. As resistance to these partner drugs starts to evolve, more pressure is placed on the artemisinin component to ensure that the ACT remains effective. Clearly, combination drugs with novel components are necessary. Artemisinins target most of the stages targeted by partner drugs (trophozoites and schizonts), but additionally, they also act against ring stages. They also have marked differences in their potencies against different asexual blood stages (see discussion of the hypersensitive profile, below) (Fig. 1). The other key difference is that artemisinins have relatively short half-lives, resulting in their presence at active concentrations for only ∼4 to 6 h posttreatment (15). Patients often present for treatment with their infections semisynchronized around a mean developmental age of typically ∼5 h (e.g., see reference 14). In these circumstances, the stage specificity of drug action has an important impact: if a patient presents with parasites in stages highly sensitive to artemisinin, then the drug will have a large effect. Conversely, if a patient has parasites that are predominantly in less sensitive stages, then the artemisinin drug action will be severely compromised.

Several studies have used pharmacokinetic/pharmacodynamic models that include more than one parasite stage (26–30). However, to our knowledge, there has been no comprehensive evaluation of the consequences of assuming parasite homogeneity in conventional continuous-time models. Heterogeneity cannot be captured by the conventional ODE approach based on a single compartment for parasite burden in red blood cells, so the established method to investigate malaria heterogeneity and drug stage specificity is to replace the continuous-time/ODE approach with a discrete-time model using difference equations (6). This approach, first described by Hoshen et al. (6) and used by others (14, 15, 31), can be briefly summarized as follows: the model tracks malaria infection by dividing parasite development within RBCs into 48 “age bins,” with each bin representing 1 h of development. These discrete-time models therefore require that each patient's treatment be described by 48 equations, each of which has to be updated for each hour of patient follow-up after treatment (typically up to 63 days [32]). While discrete-time models properly incorporate parasite heterogeneity in malaria infections, they are computationally more demanding. Furthermore, they have been described in principle (6), but to date, there appears to have been no clear investigation of how they should be applied in practice for simulation of mass malaria treatment used to optimize deployment practices (e.g., alternating deployment scenarios such as age- or weight-based dosing bands or the impact of poor patient compliance in tens of thousands of malaria patients [13]).

The objectives of this study are therefore as follows. The first objective was to investigate the validity of previous models of antimalarial drug treatment that used the continuous-time approach and therefore accepted the inherent assumptions of parasite homogeneity (e.g., see references 5, 7–13, 18, and 33). The second objective was to quantify how much more accurate and/or less biased discrete-time approaches are and to identify their appropriate calibration from clinical, field, and laboratory studies. The third objective was to identify computational shortcuts that improve the accuracy of the continuous-time approach, as the discrete-time approach is relatively slow even using modern supercomputers, so a faster continuous-time approach may provide rapid analyses appropriate in most research environments.

## MATERIALS AND METHODS

For clarity, the methods are presented in a qualitative, intuitive manner so that the concepts are, hopefully, accessible to nonmodelers. The strategy is to compare and reconcile the continuous-time and discrete-time approaches by altering the parasite killing rates to match predicted parasite numbers between the two approaches. For simplicity, we give details on monotherapy only; a discussion of how individual drug calibrations can be combined for combination therapies can be found elsewhere (12). We assume that drugs have either long or short half-lives and either do or do not have stage-specific killing. We look at all combinations, giving four drug types in total:

- “Hypothetical drug 1,” with a long half-life and without stage-specific killing.
- An ACT “partner drug,” with a long half-life and stage-specific killing. Typical examples are mefloquine and lumefantrine (killing in age bins 18 to 40 inclusive) as well as piperaquine (killing in age bins 12 to 36 inclusive) (15).
- “Hypothetical drug 2,” with a short half-life and without stage-specific killing.
- An “artemisinin derivative,” with a short half-life and stage-specific killing.

The two hypothetical drugs have properties that do not match any existing antimalarial drugs but are investigated for several reasons. First, hypothetical drugs are investigated to understand and illustrate the general principles underlying the treatment dynamics. Second, novel antimalarial drugs that have these characteristics may eventually be developed. Third, the methodology is not restricted to malaria: in principle, it can be used as a general model for treatment of infectious agents with stage specificity.

The continuous-time and discrete-time approaches must be reconciled so that they yield the same observed killing rates (quantified as the parasite reduction ratio [see the supplemental material]). All calculations were performed by using the R statistical software package (version 3.1.1) (34).

### Continuous-time models.

The basic method is based on ODEs and is widely applied for simulating antimicrobial drug treatment (see reference 35 for a review). For malaria, an ODE is used to track the change in parasite number according to the amount of drug present, i.e.,

(1) $$\frac{dP}{dt}=P\left[a-f\left(I\right)-f\left(C\right)\right]$$

where *P* is the number of parasites in the infection; *t* is time after treatment; *a* is the parasite growth rate (here we assume that each schizont releases 10 merozoites that successfully reinvade RBCs, giving an *a* value of 0.048 per 48 h); *f*(*C*) is drug parasite killing, which depends on the drug concentration, *C*; and *f*(*I*) is the killing resulting from hosts background immunity. The critical point to note is that *P* in equation 1 does not distinguish between parasite developmental stages (which we term age bins [see below]), so this standard methodological approach cannot explicitly account for stage-specific drug action. The number of parasites at time *t* after treatment (*P_t_*) is obtained by using conventional calculus as

(2) $$P_{t}=P_{0}\;e^{at}\;e^{-\overset{t}{\underset{0}{\int }}f\left(C\right)dt}$$

where *P*_0_ is the number of parasites at the time of treatment, i.e., *t* = 0 (for details on how this equation is derived, see, for example, the supplemental material in reference 11). If the minimum predicted number is <1, then the infection is assumed to be cleared.

The drug killing function, *f*(*C*), usually follows the Michaelis-Menten equation, i.e.,

(3) $$f\left(C\right)=V_{\max\;}\left[\frac{{\left(C_{t}\right)}^{n}}{{\left(C_{t}\right)}^{n}+{{\text{IC}}_{50}}^{n}}\right]$$

where *C_t_* is the drug concentration at time *t* (for details, see reference 12), *V*_max_ is the maximal drug kill rate per hour or per day, IC_50_ is the concentration at which 50% of maximal killing occurs, and *n* is the slope of the dose-response curve. Two factors determine drug killing after treatment for each drug type: its specific pharmacodynamic profile (Fig. 1) and its Michaelis-Menten function. The amount of drug killing plateaus at high concentrations at *V*_max_ (equation 3), so a useful simplification (relaxed in Section 4 in the supplemental material) is to assume that the drugs are either present and killing at maximal effect (i.e., *V*_max_) or present at negligible concentrations (i.e., essentially absent). This simple presence-absence assumption seems appropriate for the partner drugs because their long half-lives mean that they are likely to be present at high concentrations over the period of the stage-specific simulations, typically 4 days (96 h). In the case of drugs with very short half-lives, such as artemisinins, we simply define a duration of activity posttreatment (the default value being 6 h [15]). This allows the continuous- and discrete-time approaches to be matched simply by specifying a duration of time that the drug is present (and killing at maximal effect) posttreatment and matching *V*_max_ in the continuous-time methodology (equation 3) to its discrete-time counterpart, *V*′_max_ (see discussion of equation 4, below): this matching will therefore enable the continuous- and discrete-time models to be directly compared.

### Discrete-time models.

Parasites exposed to drug treatment may be in any stage of development within their 48-h life cycle in RBCs and hence differ in their sensibility to the drugs. A conventional method for dealing with such continuous data is by splitting the data into a computationally manageable number of discrete “bins.” In principle, there can be any number and length of bins in the discrete-time model, but here, according to methods described by Hoshen et al. (6), we use a simple linear approach and split the 48-h parasite development cycle in RBCs into 48 1-h bins. We refer to these entities as “bins” or “age bins” interchangeably depending on the context and need for clarity (note that Hoshen et al. [6] refer to them as “boxes”). Patients may present for drug treatment with parasites in an infinite variety of distributions among these 48 bins. If drugs preferentially act against certain age bins in the 48-h cycle, then the distribution of parasites among the age bins at the time of treatment may have an impact on the subsequent dynamics of parasite clearance. Consequently, each patient must have his/her distribution of parasites among age bins defined at the time of treatment. For illustrative purposes, we identify five “paradigm distributions” (PD1 to PD5; see Section 1 in the supplemental material) of infections that differ in distributions at the time of the start of treatment. Briefly, these are as follows:

- PD1, asynchronous and equally distributed over all age bins
- PD2, mainly in early ring stages with a relatively tight distribution across age bins
- PD3, mainly in early ring stages with a relatively wide distribution across age bins
- PD4, mainly in the late ring stages with a relatively tight distribution across age bins
- PD5, mainly in trophozoite stages with a relatively tight distribution across age bins

The first step is to define a “pharmacodynamic profile” for each drug that specifies its parasite killing for each 1-h age bin (Fig. 1). We then combine the duration of drug killing after treatment with the drug's pharmacological profile to identify a value for the maximal drug killing rate, *V*′_max_. These calculations are provided in Sections 2 and 3 in the supplemental material and are summarized in Table 1 . The killing in each age bin, *b*, at time *t* is then given as

(4) $$V_{\max\;}^{\;b,t}=Y_{b}\;Z_{t}{V\prime }_{\text{max\&ApplyFunction;}}$$

where *Y_b_* is the pharmacodynamic profile so that, in the simplest case, *Y_b_* equals 1 if the drug kills parasites in age bin *b* and *Y_b_* equals 0 if it does not kill parasites in that age bin. *Z_t_* tracks the drug concentration posttreatment so that *Z_t_* equals 1 if the drug is present at time *t* and *Z_t_* equals 0 if the drug is not present. This allows the proportion of parasites in age bin *b*, at time *t*, that survive the subsequent hour to be calculated as

(5) $$\Psi^{b,t}=e^{-V_{\max\;}^{\;b,t}}$$

which is used in equations 6 and 7 below to track parasitemia.

A two-dimensional matrix, the “parasite matrix” (PM), tracks the total number of parasites in each bin for each hour posttreatment. The first column (*t* = 1) of the PM holds the initial age bin distribution of parasites at the time of treatment. The algorithm then simply tracks the number of parasites in the 48 bins after treatment using the standard index methodology dating back to the study by Hoshen et al. (6) and subsequent studies (e.g., see references 14, 15, 17, and 31); i.e., for every age bin (*b*) at each time (*t*) posttreatment, the algorithm calculates how parasites survive drug treatment and then moves the survivors on an hour into the next age bin (i.e., *b* + 1) and into the next time period posttreatment (i.e., *t* + 1), i.e.,

(6) $${\text{PM}}_{b+1,t+1}={\text{PM}}_{b,t}\;{\text{Ψ}}^{b,t}$$

Note that for *b* = 1, we allow for the production of new parasites at the end of age bin 48, i.e.,

(7) $${\text{PM}}_{1,t+1}={\text{PM}}_{48,t}\;\Psi^{b,t}\text{ PMR}$$

where PMR is the parasite multiplication rate, i.e., the average number of merozoites released from a schizont that successfully infect new RBCs.

**TABLE 1:** Drug killing rates for the continuous-time and discrete-time models^*a*^

Drug	Half-life	Stage specificity	Continuous-time model	Discrete-time model
Hypothetical drug 1	Long	No	*V*_max_ = ln(PRR_48_)/48 + *a*	*V*′_max_ = ln(PRR_48_)/48 + *a*
Partner drug	Long	Yes	*V̂*_max_ = ln(PRR_48_)/48 + *a*	*V̂*′_max_ = *V̂*_max_ 48/*q*
Hypothetical drug 2	Short	No	*Ṽ*_max_ = [ln(PRR_48_) + 48*a*]/*t_a_*	*Ṽ*′_max_ = [ln(PRR_48_) + 48*a*]/*t_a_*
Artemisinin derivative PRR_48_ calibration	Short	Yes	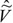 _max,48_ = [ln(PRR_48_) + 48*a*]/*t_a_*	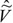 ′_max,48_ = 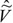 _max,48_ 48/*q*
Artemisinin derivative PRR_96_ calibration	Short	Yes	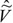 _max,96_ = [ln(PRR_48_) + 96*a*]/3*t_a_*	Obtained by iteration

a *a* is the instantaneous parasite growth rate over the 48-h parasite RBC cycle, PRR_48_/PRR_96_ is the reduction in parasite number over 48 or 96 h (i.e., one or two parasite RBC cycles) following drug treatment (the value is different for each drug but identical for both models when used for the same drug), *q* is the number of 1-h bins during which killing occurs, and *t_a_* is the duration of drug action after each dose. Shown are the equations required to convert the discrete-time model to its continuous-time equivalent for a single patient, i.e., to match the maximal parasite kill rate (*V*_max_ in equation 3) in the instantaneous model to its equivalent *V*′_max_ value in the discrete-time model (equation 4), the latter being denoted by the prime symbol. The circumflex or tilde above *V*_max_ indicates whether adjustment has been made for the effects of stage specificity or short half-life, respectively, to compensate for the lack of drug killing in nonsensitive stages and times when the drug is not present during the 48-h (or 96-h) census period.

### Reconciling the continuous- and discrete-time approaches.

The calibration requires that equivalent killing rates are identified, i.e., *V*_max_ in equation 3 and *V*′_max_ in equation 4, so that parasite numbers obtained from the continuous- and discrete-time methodologies match at the end of each 48-h cycle (see below). The values of *V*_max_ used in the continuous- and discrete-time methodologies are distinguished by using a prime symbol for the latter, i.e., *V*′_max_. A circumflex above *V*_max_ (*V̂*_max_) indicates that an adjustment has been made for the effects of stage specificity and the lack of drug killing in nonsensitive stages. A tilde above *V*_max_ (*Ṽ*_max_) indicates that an adjustment has been made for the short half-life of the drug and the times when the drug is absent (and hence not killing) during the 48-h (or 96-h) census period.

The parasite reduction ratio (PRR) is conventionally measured in the clinic as the number of (observable) parasites present at the time of treatment divided by their number 48 h later. The continuous- and discrete-time models can be calibrated by using PRR as a metric of drug killing by making allowances for the drug's half-life and the susceptible parasite age bins. The basic equations are given in Table 1, which shows how the kill rate calibrations depend on the amount of drug killing (i.e., PRR), the duration posttreatment that the drug is active, and parasite growth rate, *a*. In the case of discrete-time modeling, it also captures the number of age bins in which killing occurs (*q*).

A problem arises with the “artemisinin drug,” as it is impossible to match 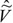
_max,48_ and 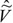
′_max,48_ such that continuous- and discrete-time models give identical parasite numbers at the end of each 48-h cycle (see below). This mismatch arises because the age bin distribution at the time of treatment has a large effect on subsequent dynamics, so 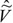
_max_ and 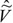
′_max_ had to be matched by using the parasite reduction ratio predicted to occur over 96 h (PRR_96_), i.e., the number of parasites present at the time of treatment divided by the number 96 h later. The calculations required for this are given in Section 3 in the supplemental material.

### Parameterization of models.

We used previously reported results where available and attempted to identify plausible values otherwise. In all cases, we use rather than endorse these calibrations, so this approach makes it straightforward for readers to calibrate the simulations according to their own local clinical and epidemiology settings.

### Simulation of artemisinin treatment in patient populations using continuous-time models.

The methods described above allowed us to calibrate the continuous-time method such that it captures the effects of stage specificity. The obvious practical application of the new methodology is to simulate the deployment of ACTs for mass treatment of patients and to assess the impact of stage specificity on predicted population-wide drug effectiveness; the latter has been missing from previous analyses. This source of variation has not been incorporated into previous simulations of ACT treatment (e.g., see references 11 and 12), so we need to incorporate and assess its likely impact on the predicted treatment outcomes. We do this by rerunning our previous simulations of artemether-lumefantrine (AM-LF) and artesunate-mefloquine (AS-MQ) treatment (12). The process for doing so is described in Section 3 in the supplemental material. In brief, we ran the model for multiple patients to determine the population PRR_96_ and used this to obtain a continuous-time approximation for 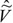
′_max,96_. This new estimate of 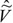
′_max,96_, and its associated interpatient variability, was then incorporated into mass simulations of ACTs to account for the stage-specific effects of the artemisinin component.

## RESULTS

### Continuous-time and discrete-time models for different types of drugs.

**FIG 2:**
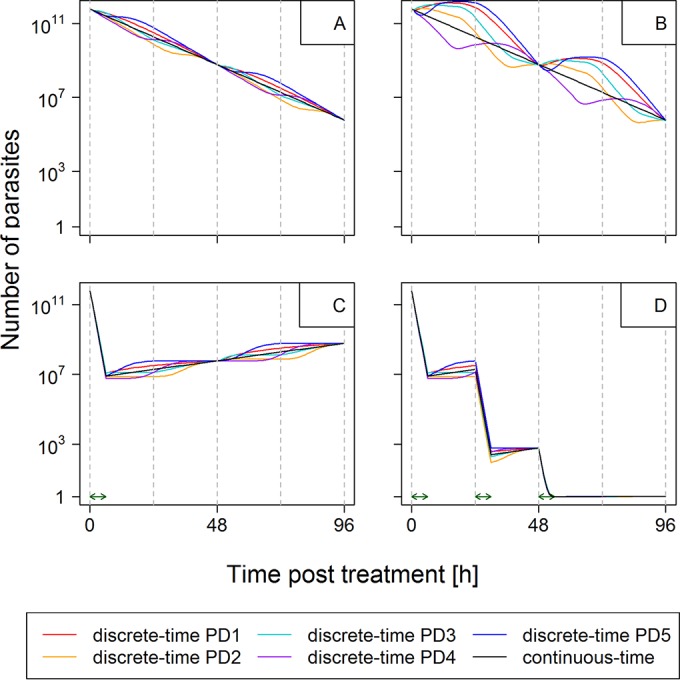
Changes in parasite numbers following treatment. The graph shows the number of parasites over time posttreatment. Parasites present at the time of treatment were distributed among age bins according to PD1 to PD5 (see Section 1 in the supplemental material). Note that the number of parasites is the true number, i.e., circulating plus sequestered, plus 1 [it is conventional to plot parasites + 1 when using a log scale because log(0) is undefined]. (A) Drug with a long half-life and equal killing in all age bins (e.g., hypothetical drug 1). This was produced by using the pharmacodynamic profile of hypothetical drug 1. The discrete-time model used a drug killing rate, *V*′_max_, of 0.1919 and a *Y_b_* of 1 for age bins 1 to 48, and the continuous-time model used a drug killing rate, *V*_max_, of 0.1919. (B) Drug with a long half-life and stage-specific killing (e.g., lumefantrine). This was produced by using the pharmacodynamic profile of the drug lumefantrine. The discrete-time model used a drug killing rate, *V̂*′_max_, of 0.4005, a *Y_b_* of 1 for age bins 18 to 40 inclusive, and a *Y_b_* of 0 for age bins 0 to 17 and 41 to 48 inclusive, and the continuous-time model used a drug killing rate, *V̂*_max_, of 0.1919. (C) Drug with a short half-life and equal killing in all age bins (i.e., hypothetical drug 2), given as a single dose and assuming that the drug is present and acting at maximal killing for 6 h posttreatment (15). The discrete-time model used a drug killing rate, *Ṽ*′_max_, of 0.1919; a *Y_b_* of 1 for age bins 1 to 48; and a *Z_b_* of 1 for the 6 h that the drug was present, and the continuous-time model used a drug killing rate, *Ṽ*_max_, of 1.919 for a single dose administered at time zero (green arrow). (D) Same as for panel C but with three doses administered at 0, 24, and 48 h (green arrows).

The parasite numbers predicted by the continuous-time and discrete-time models for a drug with a long half-life that kills all parasite stages (hypothetical drug 1) are compared in Fig. 2A. The lack of stage-specific killing means that variation around the continuous-time approximation is due solely to differences caused by parasites reproducing at the end of their 48-h cycle. Infections that were initially in late age bins, such as PD5, will rupture and produce new parasites (merozoites) early in the 48-h census period, so parasite numbers will remain higher than the continuous-time prediction over most of the census period. Those infections that were initially in early age bins of the cycle, such as PD2, release merozoites late in the 48-h census period, so their numbers will usually lie below the continuous-time approximation. As expected, all predicted numbers converge to the same value at the end of each 48-h census period.

Figure 2B compares parasite numbers predicted by the continuous-time and discrete-time models for a drug with a long half-life that has stage specificity. The example shown in Fig. 2B is for the “lumefantrine” pharmacodynamic profile, but similar results were obtained for the “piperaquine” profile (see Fig. S3 in the supplemental material). The major difference between the data in Fig. 2A and B is that in Fig. 2B, the effect of stage specificity is added to the effect of initial age bin distributions, and the variation around the continuous-time approximation is substantially increased compared to that shown in Fig. 2A. The patterns of variation can be understood as the interaction between these two effects. In an infection with parasites that are predominantly in late age bins at the start of treatment (e.g., PD5), some parasites are killed, but many parasites survive to rupture and release merozoites that are then unaffected by the drug for the next 18 h (Fig. 1). Consequently, parasite numbers in an infection with PD5 stay well above the continuous-time approximation for the whole census cycle. When parasites are mainly in early bins (e.g., PD2) at the time of treatment, they are not affected by the drug, and their total number is initially above the approximation until the time point when the parasites start to enter the sensitive bins (at 18 h), where intense killing brings their total number down below the number predicted by the continuous-time model. Parasites initially distributed according to PD4 suffer badly from both effects, as their mean age is 20.5 h; i.e., parasites are initially killed very effectively by the drug, and only when significant rupture and release of merozoites occur at around 20 h posttreatment does their number start to reconverge toward that predicted by the continuous-time model.

Figures 2C and D compare parasite numbers predicted by the continuous-time and discrete-time models for a drug with a short half-life that kills all stages (i.e., hypothetical drug 2). The major difference between data in Fig. 2A (hypothetical drug 1) and those in Fig. 2C and D is that hypothetical drug 2 persists for only a relatively brief period after treatment. The short half-life means that such drugs would probably be given repeatedly, so the dynamics are shown for both a single dose (Fig. 2C) and three repeated doses (Fig. 2D). Parasite numbers initially fall rapidly, and their subsequent recovery is then driven by the same dynamics as those for longer-half-life drugs without stage specificity (Fig. 2A); i.e., parasite numbers in PDs with a high mean (e.g., PD5) multiply sooner in the 48-h census period and are thus usually higher than predicted by continuous-time models, while those in PDs that have a low mean (e.g., PD2) multiply later in the 48-h census and are thus usually lower than predicted. Critically, all PDs and the continuous-time approximation reconverge at the end of each 48-h cycle.

**FIG 3:**
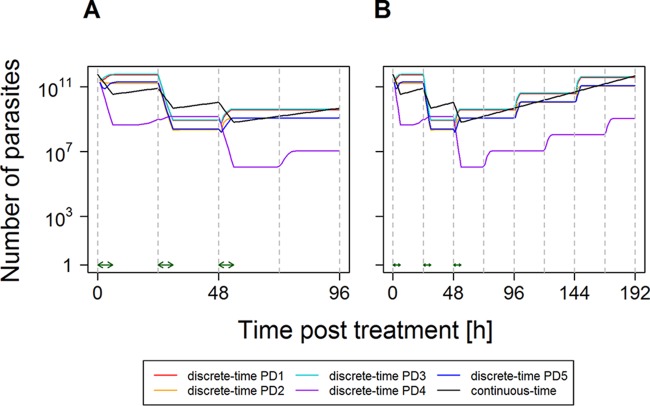
Changes in parasite numbers following treatment by a drug with a short half-life and stage-specific killing (e.g., artemisinin derivative). This was produced by using the isosensitive pharmacodynamic profile of the artemisinins (Fig. 1) and assuming that the drug is present and acting at maximal killing for 6 h after each dose (15). Artemisinins are simulated as a monotherapy for clarity. They can later be combined to simulate combination therapies (12), so parasite numbers start to increase shortly after the final dose. Parasites present at the time of treatment were distributed among age bins according to PD1 to PD5 (see Section 1 in the supplemental material). The continuous-time model used a single-drug killing rate, 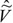
_max_, of 0.52408, i.e., the one calibrated to give a PRR_48_ of 10^4^ for a uniform distribution (Table 2). Note that the number of parasites is the true number, i.e., circulating plus sequestered, plus 1 [it is conventional to plot parasites + 1 when using a log scale because log(0) is undefined]. (A) Dynamics in detail up to 96 h; (B) how parasite numbers remain separate thereafter.

**FIG 4:**
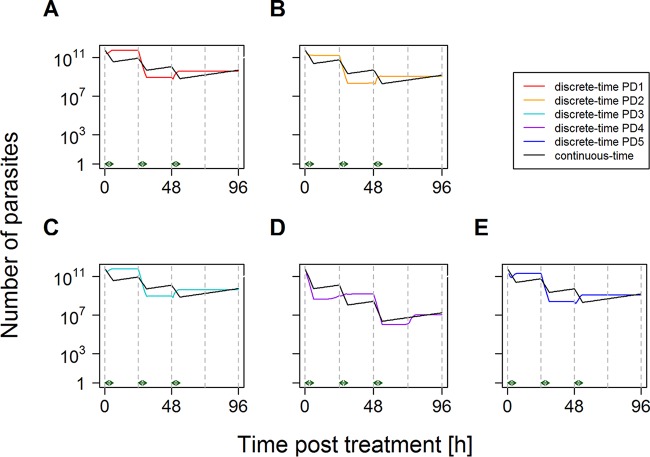
Changes in parasite numbers following treatment by a drug with a short half-life and stage-specific killing with continuous-time approximation corrected for patients' differing bin distributions at the time of treatment. This was produced by using the isosensitive pharmacodynamic profile of the artemisinins (Fig. 1) and assuming that the drug is present and acting at maximal killing for 6 h after each dose (15). Parasites present at the time of treatment were distributed among age bins according to PD1 to PD5 as described in the text. Unlike Fig. 3, the discrete-time analysis of stage specificity and its continuous-time approximation reconverge at 96 h for each paradigm distribution. The artemisinins have disappeared from the circulation by this time, so the continuous-time approximation captures the total amount of artemisinin drug killing. These examples use the continuous-time kill rate, 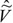
_max,96_, appropriate for each distribution (Table 2), i.e., a 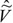
_max,96_ of 0.524 for PD1 (A), a 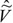
_max,96_ of 0.591 for PD2 (B), a 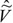
_max,96_ of 0.518 for PD3 (C), a 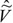
_max,96_ of 0.837 for PD4 (D), and a 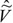
_max,96_ of 0.588 for PD5 (E). Note that the number of parasites is the true number, i.e., circulating plus sequestered, plus 1 [it is conventional to plot parasites + 1 when using a log scale because log(0) is undefined].

Figure 3 compares the continuous-time and discrete-time models for a drug with a short half-life with the stage-specific characteristics of the artemisinin class of drugs. It is extremely difficult to capture the posttreatment dynamics by a single continuous-time equation because of the impact of an infection's age bin distribution at the time of treatment. Figure 3 used the continuous-time approximation with a 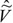
_max,48_ calibrated from PD1 (using Equation S16 in the supplemental material). Note that, for instance, PD4 is very poorly captured by this approximation, and importantly, the parasite numbers do not reconverge every cycle (Fig. 3A in contrast to 2A to D), so the mismatch will be perpetuated over subsequent cycles (Fig. 3B). This makes it necessary to use a different continuous-time calibration for each of the five paradigm distributions by using the approach leading to Equation S26 in Section 3 in the supplemental material (Fig. 4). Slight differences between the discrete- and continuous-time methods for each paradigm distribution occur, but importantly, the continuous- and discrete-time methods always reconverge after 96 h (Fig. 4), irrespective of the age bin distribution at the time of treatment (the panels in Fig. 4 illustrate five very different starting age bin distributions), and every 48 h thereafter, as shown in Fig. S4 in the supplemental material. The first convergence occurs after 96 h because parasite killing of artemisinins has to be calibrated over a 96-h period (rather than the 48-h period for the other examples). The convergence in subsequent 48-h census periods is due to the match in the PMR.

### Mass simulations of treatment.

We replicated our recent mass simulation of AM-LF and AS-MQ treatment (12) to include the stage-specific drug action of artemisinins by allowing an additional 2-fold variability around the artemisinin 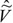
_max,96_ (see Equation S28 in the supplemental material). Its inclusion made very little difference in the results (see Fig. S5 and S6 and Table S2 in the supplemental material): cure rates using our original mean 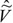
_max,96_ of 27.6 per day changed from 84.74% to 84.13% for AS-MQ and from 92.29% to 91.76% for AM-LF. There was similarly a very small effect of stage specificity when we reduced the artemisinin 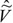
_max,96_ to 14.6 per day (the reasons for using this lower artemisinin 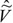
_max,96_ are explained below).

## DISCUSSION

### Comparison of outputs from continuous-time and discrete-time models for different types of drugs.

The calibrations presented in the supplemental material and summarized in Table 1 enabled the continuous- and discrete-time methods to be calibrated in an equivalent manner. This allowed us to investigate the extent to which the continuous-time approximation captures the more biologically realistic discrete-time models.

Initial investigations used the simplest example of hypothetical drug 1, which is assumed to have a long half-life and kill all age bins. This isolated the effect of replication at the end of the RBC life cycle to be the only difference between the continuous- and discrete-time approaches. Results suggest that replication solely at the end of the 48-h cycle introduced only a small amount of variation around the treatment dynamics predicted by a continuous-time approach (Fig. 2A). The discrepancy between predicted and actual numbers is small, about plus/minus half a log_10_ unit, and, importantly, is constant over subsequent cycles. The latter point is important because the infection is deemed to have been cleared if the expected number of parasites falls below 1, and the variation around the predicted parasite number at that point is relatively low, suggesting that the continuous-time approximation for therapeutic outcome (i.e., cure/fail) should be applicable for this type of drug. Our (subjective) interpretation of these results is that the assumption of continuous replication is unlikely to have a significant impact on the results from studies where drugs lack stage-specific activity.

The next step was to add stage-specific drug action to a long-half-life drug (i.e., the ACT partner drugs). This combined the impact of stage specificity with that of replication occurring only at the end of the 48-h life cycle. The results are illustrated in Fig. 2B. As might be expected, stage specificity introduces considerably more variation around the continuous-time approximation. These are important examples, as they characterize an antimalarial “partner” drug whose treatment has been previously examined by using a continuous-time approach by both us (e.g., see references 11–13) and others (e.g., see references 7, 10, and 33). An important, and long overdue, question is the extent to which the continuous-time approach truly predicts the drug posttreatment parasite dynamics. We would argue, again subjectively, that the approximation is good. The key factors are that the variation disappears every 48 h and that it scales with parasite number such that the maximum deviation is around 2 log_10_ units, i.e., a factor of 100. The continuous-time approach defines the infection as “cured” when the predicted number of parasites falls below 1. Figure 2B and Fig. S3 in the supplemental material suggest that this may arise if the predicted number was within 2 log_10_ units on either side, i.e., from 0.01 to 100. It seems intuitively likely that discrepancies of this relatively small magnitude would rarely occur and, consequently, that continuous-time simulations would be accurate. This argument also assumes the worst-case scenario, i.e., that the drug instantaneously disappears at exactly the point when the discrepancy is maximal. In reality, the smooth transition from maximum killing to ineffective concentrations would likely help smooth out the discrepancies.

The third drug class investigated was drugs with a short half-life and without stage-specific killing (i.e., hypothetical drug 2). The short half-life means that parasite numbers initially fall rapidly but recover once the drug is not present anymore (Fig. 2C and D). The change in parasite number is driven by the same dynamics as those of longer-half-life drugs without stage specificity (Fig. 2A), and the continuous-time approximation reconverges at the end of each 48-h cycle. This reconvergence plus the relatively small deviations between the model types suggest that, should such an antimalarial be discovered and deployed, the continuous-time methodology would be an appropriate simulation method.

Finally, the effects of short half-life, stage-specific killing, and replication only at the end of the 48-h cycle were investigated (i.e., the artemisinin derivatives). The implications are much more serious for the continuous-time approach. Figure 3 shows the dynamics of artemisinin treatment: the deviation from the continuous-time approximation is larger, e.g., ∼3 log_10_ units or 10^3^-fold in the case of PD4, and critically, the deviation does not periodically disappear (as it does every 48 h for partner drugs) (Fig. 2B; see also Fig. S3 in the supplemental material). Consequently, deviations persist over time and will plausibly have an impact on the predicted therapeutic outcome. In our opinion, this is an unacceptable level of divergence, and we conclude that artemisinin treatment cannot be adequately modeled in the same way as the other drugs because the initial age bin distribution at the time of treatment has such a large effect on the PRR.

Figure 4 shows that a continuous-time approximation calibrated for initial bin distribution accurately tracks killing over the two 48-h parasite life cycles that artemisinins are present and supports our assertion that the use of infection-specific continuous-time kill rates, 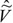
_max,96_ (see Fig. S7 in the supplemental material), can capture the variation introduced into posttreatment dynamics by patients' differing age bin distributions at the time of treatment. The essence of our argument is that the effects of differing bin distributions at the time of treatment can be incorporated simply by inflating the variation in a drug's maximal kill rates.

### Estimates of artemisinin kill rates.

The inclusion of stage specificity into our recent mass simulation of AM-LF and AS-MQ treatment (12) made very little difference in the results (see Fig. S5 and S6 and Table S2 in the supplemental material). There was similarly a very small effect of stage specificity when we reduced the artemisinin 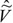
_max,96_ to 14.6 per day (the reasons for investigating this reduced 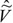
_max,96_ are explained below). The analyses show that artemisinin kill rates (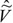
_max,96_ of ∼0.6 per h) (Table 2; see also Fig. S7 in the supplemental material) are much lower (by a factor of ∼2) than estimated in our previous studies, which used values of 27.6 per day (12, 13), equivalent to 1.15 per h (i.e., 27.6/24). There appear to be two underlying reasons for this: one is the use of the PRR to calibrate the killing and the other is the extrapolation of the PRR to overall kill rates (each is discussed below).

**TABLE 2:** Impact of age bin distribution at time of treatment on continuous-time artemisinin kill rates^*a*^

Distribution (mean, SD [h])	True PRR_48_	Apparent PRR_48_	True PRR_96_	Apparent PRR_96_	Kill rate ( 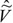 _max,96_)
PD1 (uniform)	541	10,054	125	14,268	0.52408
PD2 (10.5, 5)	2,032	20,024	416	34,692	0.59085
PD3 (10.5, 10)	518	11,873	112	17,533	0.51776
PD4 (20.5, 5)	324	84,293	34,822	8,770,475	0.83684
PD5 (35.5, 5)	1,889	3,069	397	3,145	0.58822

a The true PRR is the reduction in the total number of parasites, and the apparent PRR is the reduction in observable (i.e., nonsequestered and, thus, circulating) number of parasites per 48 or 96 h. A discrete-time artemisinin kill rate (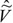
′_max,48_ = 1.164) was obtained, which gave an apparent parasite reduction ratio (PRR_48_) of ∼10^4^ (actually 10,054) by using the following assumptions: (i) there is a uniform age bin distribution, (ii) three doses of an artemisinin are given at 0, 24, and 48 h (although, obviously, only the first two doses contribute to the PRR_48_) and persist for 6 h following each dose, (iii) there is an isosensitive pharmacodynamic profile (14), and (iv) parasites immediately disappear from the circulation at age bin 14 (see the supplemental material for methodological details and Table S1 in the supplemental material for more results). The continuous-time-equivalent artemisinin drug kill rate (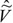
_max,96_) is calculated from the true PRR_96_ by using Equation S26 in the supplemental material. Note that the discrete-time kill rates are identical for each row (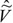
′_max,48_ = 1.164) so that the variation in the continuous-time kill rate (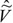
_max,96_) is caused solely by the differences in age bin distribution at the time of treatment. The dynamics of treatment are shown in Fig. 4.

Previous simulations of artemisinin treatment were calibrated by using the observed PRR (i.e., the reduction in circulating and sequestered parasites) of ∼10^4^ as reported in the literature and defined as the reduction in the number of parasites observed in the peripheral blood by microscopy. This is potentially misleading because it does not capture changes in the number of sequestered parasites. Our simulations allow us to calculate both “apparent” and “true” PRRs and suggest that the apparent PRR_48_ is substantially higher than the true PRR_48_ (Table 2). The effect of short pulses of stage-specific artemisinin killing on observable, circulating parasites (age bins up to 14) and sequestered parasites (age bins 15 and above), and, hence, on the observed PRR, varies greatly depending on the initial age bin distribution of the parasites (see Fig. S10 and S11 in the supplemental material).

The second factor behind the discrepancy in artemisinin maximal kill rates arises because, *in vivo*, the PRR is typically measured over 48 h. This omits the impact of the final dose at 48 h, and it is assumed that the results for the first two doses (which determine the PRR) may be extrapolated for the third dose. However, a dose of artemisinin given 48 h after the first dose will affect exactly the same age bins already targeted by the first dose. Consequently, this third dose is likely to have much less of an impact than the first two doses. Calibration against PRR_48_ captures the effects of only the first two doses and will thus overestimate the impact of the third dose. Calibration against PRR_96_, as done here, incorporates the reduced impact of the third dose, and so the estimated artemisinin kill rates, 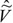
_max,96_, are further reduced.

As may be expected, this reduction in the artemisinin kill rate may have a significant impact on simulated drug effectiveness. Our mass simulations based on previous work (12) show that reducing the 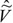
_max,96_ from 27.6 to 14.4 per day (i.e., 24 × 0.6 = 14.4 to convert hourly to daily kill rates) roughly doubled the number of predicted treatment failures (see Table S2 in the supplemental material).

### Impact of stage specificity on mass simulations of ACT treatment.

Incorporation of the 2-fold variation caused by age bin distributions again had a negligible effect, as seen with the higher kill rate. The underlying reason appears to be that this 2-fold variation adds very little to the natural variation in parasite sensitivity to the drug's 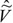
_max,96_, whose coefficient of variation (CV) was assumed to be 0.3 (12) (this is shown in Fig. S5 and S6 in the supplemental material). Recall that we first sampled 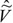
_max,96_ from a normal distribution to reflect the natural variation among parasites in their 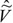
_max,96_ values; the resulting simulated distributions are shown as rows A and C in Fig. S5 and S6 in the supplemental material. We then resampled 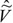
_max,96_ from a 2-fold range around this selected value to allow for differences in the age bin distribution of infections at the time of treatment (see Fig. S7 in the supplemental material); the distributions of these resampled values are shown in rows B and D in Fig. S5 and S6 in the supplemental material. Note that the variation increases slightly as this 2-fold effect is included and that the distribution becomes slightly more right-skewed. The skew arises because the uniform distributions are scaled against the selected value of 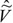
_max,96_ (see Equation S28 in the supplemental material), so high values (at the right-hand side of the distribution) have higher additional variation that tends to slightly skew the distribution at this side. The important point is that the variation in 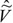
_max,96_ values increases only marginally in rows A and C versus rows B and D in Fig. S5 and S6 in the supplemental material. In effect, it appears that the additional variation introduced by artemisinin stage-specific killing and its short half-life is largely incorporated into the natural background version in 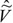
_max,96_ so that the impact on cure rates, at least in our examples, is negligible (see Table S2 in the supplemental material).

Variation in age bin distributions at the time of treatment therefore appears to have little impact on our simulations, but there is no guarantee that this will be the case in all studies, and it is good practice to incorporate this effect if possible. The results shown in Fig. S7 in the supplemental material suggest a general rule of thumb: in the absence of any better information, the natural variation in the artemisinin kill rate, 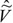
_max,96_, should be augmented 2-fold to incorporate age bin variation in patients at the time of treatment. Our mass simulation, however, showed that the addition of this variability to an individual's drug killing rate, 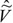
_max,96_, did not affect predicted cure rates (see Table S2 in the supplemental material). The natural variation around the mean of 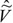
_max,96_ is so large (i.e., CV = 0.3) that the distribution of the patients' 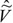
_max,96_ barely changes when the correction for stage specificity is added (see Fig. S5 and S6 in the supplemental material).

### Impact of adherence.

The simulations assumed full patient adherence to 24-h dosing intervals. However, in practice, patients may miss a dose, delay a dose by several hours, or finish treatment early. We investigated adherence in a previous report (13) but assumed that artemisinin doses were all equally effective. In reality, the impact of dose timing and the fact that the third dose of the artemisinin appears to have less of an impact suggest that a more nuanced approach could be used to investigate the impact of poor adherence. This could be incorporated in the same way as the effects of the initial bin distribution, i.e., by simulating a range of initial age bin distributions with a range of adherence patterns, computing PRR_96_ for each patient within the population, and using this to generate the distribution of 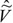
_max,96_ analogous to that shown in Fig. S7 in the supplemental material, which also incorporates the effect of adherence patterns.

### Conclusions.

The potential impact of age bin distribution on drug treatment may be obvious in retrospect. In fact, it is not a new idea but seems to have been lost in the artemisinin era (just when it was most relevant). The stage-specific action of antimalarials has been investigated since the early 1980s (21, 36, 37), so it is therefore not surprising that chronotherapy for malaria, i.e., the science of the timing of drug application so as to achieve optimal therapeutic success for the treatment of disease, is an old idea (38). Following the administration of an ACT, the partner drug is present in a patient's blood at concentrations above the MIC over several parasite life cycles of 48 h (39), so it is therefore unlikely that the timing of partner drug application would affect treatment outcome (Fig. 2B). However, the artemisinins are present in the blood at concentrations above the MIC only during a very short period of time, i.e., 4 to 6 h (15), and chronotherapeutic considerations seem justified (Fig. 3). It is difficult to envisage exactly how this would be achieved in practice (it would be unethical to delay treatment), but more frequent dosing with artemisinins, as occurs in the twice-per-day regimen of AM-LF treatment, may help in this respect and deserves further investigation. As mentioned above, the WHO recently recommended the use of mathematical models of antimalarial chemotherapy for a better understanding of drug resistance and its management (40). The advantage of mathematical models is that they can overcome some of the experimental, ethical, or logistic issues associated with *in vitro* experiments or clinical trials on the stage specificity of antimalarials.

The discrete-time methodology will remain the “gold-standard” simulation method, but we believe that continuous-time methods will continue to be used in the foreseeable future because they offer a substantial increase in computational speed with, as we show in this study, no compromise in the validity of their results. The increase in speed arises because the discrete-time models track 48 parasite developmental “bins,” each of which has to be updated every hour (i.e., 24 times per day). In contrast, the continuous-time method tracks only the total number of parasites and, for most malaria drugs, is updated only daily. The ratio of computations (and, hence, basic speed) is therefore 1:(48 × 24), making the continuous-time approach >1,000-fold faster (with the exception of artemether-lumefantrine, which is administered twice daily, in which case the computational advantage halves to ∼500-fold). Moreover, this simple calculation ignores the computational opportunity of time-saving by using calculus to project forward after the final dose in the continuous-time methods (see the appendix in reference 7). In crude terms, this means that the continuous method can run overnight (half-day) what the discrete-time method would take around a year to achieve. These simulations are highly suitable for parallel or batch processing over multiple computer cores, but no matter how many batches or cores are used, the 500- to 1,000-fold speed advantage still remains. Computational speed is important because malaria simulations have grown increasingly complex to take advantage of increased computational power, and large-scale modeling is envisaged to play a significant role in optimizing malaria control and elimination programs (3). For example, we have embedded a continuous-time methodology of drug treatment into the large-scale OpenMalaria microsimulation of malaria epidemiology (e.g., see references 41 and 42). Testing of various permutations of malaria epidemiology, transmission, and clinical practices typically takes 2 to 3 weeks to complete, so computational speed remains a priority in such situations. Similarly, investigating the large number of different permutations of age- and weight-banding patterns under a variety of target dose ranges (in milligrams per kilogram of body weight) (see reference 13) is computationally intensive, and a 500- to 1,000-fold increase in speed is extremely valuable in this context. What this paper has achieved is to validate a methodology, with particular relevance for artemisinins, that offers an extremely large increase in computational speed and that confirms the validity of previous analyses using the continuous-time approach.

This piece of work is overdue and ideally would have been performed before undertaking the mass simulations of malaria treatment that ignored stage specificity (we consider ourselves as guilty as anyone in this respect). It is interesting that the sizes of the impacts of the three features of stage specificity are in reverse order of those anticipated at the start of this work. Stage specificity of artemisinin killing inflates the variance associated with treatment but is largely lost in the context of “natural” parasite variation in drug sensitivity (see Fig. S5 and S6 in the supplemental material) and had little impact on our predicted ACT effectiveness (see Table S2 in the supplemental material). Stage specificity and the long half-life of partner drugs have some impact on the minimum number of predicted parasites and, hence, the predicted therapeutic outcome, but the likely size of this effect seemed small and can be monitored by recording the minimum number of predicted parasites in each patient (see Table S2 in the supplemental material). The largest effect arose from the combination of sequestration and a reduced impact of the third dose of artemisinin. This led to the estimated artemisinin killing being around half that obtained previously from a cruder interpretation of the PRR over 48 h (i.e., assuming that all parasites are observable) and had a large impact on predicted cure rates (see Table S2 in the supplemental material). However, we stress that these are initial conclusions based on a reanalysis of some of our previous simulations of ACT treatment with the specific pharmacokinetic/pharmacodynamic calibrations described above. Our explicit objective here was to develop and present the computational techniques necessary to bring stage specificity into mass simulations of drug treatment regimens. In order to maintain a publication of manageable size, we chose not to undertake a systematic investigation of parameter space. We have attempted to be as transparent and flexible as possible so that users can easily calibrate and apply the techniques to their own particular settings and simulations. We strongly recommend that stage specificity be explicitly considered in simulations of malaria treatment and look forward to the results obtained from other studies.

## Supplementary Material

Supplemental material
