# Supplementary material for: Incorporating Stage-Specific Drug Action into Pharmacological Modeling of Antimalarial Drug Treatment
Source: Antimicrob Agents Chemother. 2016 Apr 22;60(5):2747–56. doi: 10.1128/AAC.01172-15 (PMC4862506; doi:10.1128/AAC.01172-15)
Supplement: Supplemental material [file supp_60_5_2747__index.html]

Supplemental material 

# Incorporating Stage-Specific Drug Action into Pharmacological Modeling of Antimalarial Drug Treatment

## Supplemental material

- Supplemental file 1 -

  Supplemental Text, Tables S1 and S2, and Figures S1 to S11.

  PDF, 1.5M
