# Supplementary material for: Incorporating Stage-Specific Drug Action into Pharmacological Modeling of Antimalarial Drug Treatment
Source: Antimicrob Agents Chemother. 2016 Apr 22;60(5):2747–56. doi: 10.1128/AAC.01172-15 (PMC4862506; doi:10.1128/AAC.01172-15)
Supplement: Supplemental material [file AAC.01172-15_zac005165105so1.pdf]

1. Parameters and definitions

Initial parasite number

The initial total parasite number used in each simulation was 6×10^{11}. This is an arbitrary number, which does not affect the methodology, and represents a rather intense symptomatic infection.

Paradigm distributions (PD) of parasites

For illustrative purposes we specify the initial number of parasites present at time of treatment and their distribution across the 48 bins in the discrete-time model, i.e. PD1–5, as follows:

- PD1 is a uniform distribution where parasites are equally distributed across all the 48 hourly age-bins. This may represent a long-established infection in a high transmission area where patients are often semi-immune and asymptomatic, several malaria clones may be present, and synchronicity has been largely lost over the course of the infection.
- PD2 is an infection where most parasites are early ring stages with age-bin mean = 10.5 hours and standard deviation (SD) = 5 hours. This paradigm was based on Saralamba *et al*. (1) where the mean age was 4–16 hours and the SD was 2–8 hours. It has been argued that this may represent a relatively common situation in non-immune patients whose infected RBCs have ruptured (i.e. reached the end of their 48-hour cycle) during the night causing fever and driving the patient to seek treatment early next day (2).
- PD3 is as for PD2 but has greater variation, i.e. age-bin mean = 10.5 hours and SD = 10 hours.
- PD4 represents an infection where most parasites are in the middle of their 48-hours life-cycle, i.e. age-bin mean = 20.5 hours and SD = 5 hours.
- PD5 represents an infection where most parasites are more advanced in their 48-hours life-cycle, i.e. age-bin mean = 35.5 hours and SD = 5 hours.

Note that the normal distribution is retained across the boundary between bins 48 and 1; as an example when mean = 5.5 hours, parasites which are 6 hours younger the mean are placed in bin 48, those which are 7 hours younger are placed in bin 47, and so on. In principle the distribution could have a discontinuity to allow for parasite multiplication at the 48/1-hour boundary but this seemed an unnecessary elaboration, especially because parasitaemia at time of treatment is often being regulated by host factors so that parasites numbers are being held roughly stable. Furthermore, we set the maximum width of the normal distribution, i.e. the number of age-bins above and below the mean that we wish to consider, to 15 so that the normal distributions are never assigned across more than 30 age-bins.

Parasite growth rate

Parasites that survive through their 48 hours of development in red blood cells (RBCs), i.e. reached the schizont stage, release a number of new parasites (merozoites) that may successfully invade new RBCs. The average number of merozoites per schizont that successfully infect new RBCs after every 48-hour cycle is called the parasite multiplication rate (PMR). The continuous-time models require an instantaneous growth rate, a, over the 48-hour parasites RBC cycle so

$$\text{PMR} = e^{48a}$$

Equation S1

giving

$$a = \frac{\ln(\text{PMR})}{48}$$

Equation S2

PMR is usually set at 10 for stage specific simulations (1, 3), giving *a* = 0.048. This value will be used throughout the calibrations and simulations detailed below.

Sequestration and parasite reduction ratio (PRR)

An important feature of *falciparum* malaria parasites is their ability to sequester RBCs infected with *falciparum* malaria, parasites start to sequester (i.e. bind to the endothelium of capillaries and venules) as the parasites develop and late trophozoites and schizonts are therefore not observed in the peripheral blood (4). Sequestration generally starts around 11 hours after RBC invasion and is, most plausibly, a survival strategy of the parasite to prevent it being cleared by the spleen. This has important practical consequences because peripheral blood samples taken to assess parasitaemia contain mostly young parasites (4). This becomes very important when calibrating drug kill rates against the reduction in parasite numbers observed over a 48-hour period.

The PRR_{48} is defined as the reduction in parasite number over 48 hours (i.e. one parasite RBC cycle) following drug treatment. This is the key clinical observation used to quantify drug kill rates and, according to White (5), the *in vivo* PRR_{48} is about 10^3 for partner drugs and 10^4 for artemisinins (although PRR_{48} measured *in vitro* may be higher (6)). Previous calibrations assumed this was a reduction in 'true' total parasite number, whereas in reality it is the reduction in observable, non-sequestered parasites (the 'apparent' PRR). We therefore make a distinction between the 'true' PRR, which we define conventionally as the reduction in the total numbers of parasites, and the 'observed' or 'apparent' PRR, which we define as the reduction in the number of parasites observed in the peripheral blood by microscopy. Here we assume that parasites in bins 1 to 14 are the only ones detectable and calculated observed PRR_{48} based on this assumption. We also use a more complicated algorithm in which infected RBCs gradually disappear from the circulation. Again we used a published model, that of Saralamba *et al*. (1)), who assume infected RBCs start to sequester from bins 11 hours onwards with an exponential decay (Equation S8 of Saralamba *et al.* (1)). Figure S1 shows how 'true' and 'apparent' PRR vary depending on age-bin distribution at time of treatment.

Simulated patient populations (SPPs)

Drugs deployed into the general population will be used to treat patients whose infections will differ substantially in their age-bin distributions at time of treatment. Table 2 of the main text shows that drug effectiveness is affected by parasite age-bin distributions at time of treatment. Simulations designed to test drug effectiveness after general deployment therefore

require the relative frequencies of parasite age-bin distributions in the simulated patient population (SPP) at time of treatment. Here we define three arbitrary SPPs for later use.

- SPP1 represents a high transmission clinical setting. Many infections have lost synchronicity and have a uniform age-bin distribution as in PD1 (30%). The other patients present with infections in a variety of age-bin distributions (i.e. mean age-bin 0.5–47.5 hours) with high SDs (i.e. 6–8 hours).
- SPP2 represents a clinical setting of low transmission and with good clinical care. Most infections are symptomatic so there are few uniform infections (5%). Patients at time of treatment tend to have infections with tighter distributions (i.e. SD of 2–4 hours). It has been asserted that rupture of merozoites causes fever and drives patents to seek treatment (2) so it assumed most patients present with low mean values of the parasite distribution (i.e. 0.5–16.5 hours).
- SPP3 represents a clinical setting of low transmission and with poor clinical care. The distributions are as for SPP2 except that poor access to treatment means that patients present with a much wider range of mean values (i.e. 0.5–47.5 hours).

The methodology does not rely on these exact distributions but subsequent calculations require these distributions to be defined, and SPP1-3 (at least to us) seem plausible illustrations. Readers are able, and encouraged, to define a SPP specific to their own favoured epidemiological/clinical setting.

Pharmacodynamics of drug action

Drugs that have stage specific activity require a defined ‘pharmacological profile’ that identifies how each age-bin is affected by the drug (Figure 1 of the main text). For ‘Partner Drugs’ we use the specific examples of mefloquine and lumefantrine (killing occurs only in age-bins 18 to 40 inclusive) or piperaquine (killing occurs only in age-bins 12 to 36 hours inclusive) (3). We look at two artemisinin pharmacodynamics profiles:

- An ‘iso-sensitive’ profile (based around data in (1)) that assumes that killing occurs from 6 to 44 hours inclusive and that each of these age-bins is equally drug-sensitive (although, in reality, the results presented in the supplementary information of (1) suggest earlier ring stages are less sensitive).
- A ‘hyper-sensitive’ profile (based around data in (7)) which postulates that youngest rings (2–4 hours post-invasion) are hypersensitive and that more mature ring stage (6–20 hours post-invasion) are generally rather insensitive to artemisinins. In the hyper-sensitive model we set the kill rate at 10 $V'_{\max}$ between 2 to 4 hours, $V'_{\max}$ /10 between 6 to 19 hours, $V'_{\max}$ between 20 and 44 hours and zero for hours 1, 5 and from 45 to 48 inclusive (this was achieved by giving Y_b values of 10, 1, 0.1 or 0 as appropriate in Equation 4 of the main text).

2. Calibrating equivalent continuous- and discrete-time models for drug treatment

In this section we show how both types of approach, i.e. the continuous-time and the discrete-time model, can be calibrated for antimalarial drugs allowing direct comparison between the two approaches. We explain step-by-step how the maximal kill rate can be derived for the four types of drugs with different pharmacokinetic and pharmacodynamic properties. The symbols $V_{\max}$ and $V'_{\max}$ will be used for the maximal killing rate for the continuous- and discrete-time methods respectively i.e. the prime indicates its use in the discrete-time analyses.

We initially assume the drug is either present and killing at its maximum rate $V_{\max}$, or absent. This means if drug is present the drug parasite killing $f(C) = V_{\max}$ in Equation 3 of the main text. This allows us to integrate $V_{\max}$ between time zero and time t (Equation 2 of the main text) as

$$\int_0^t V_{\max} = tV_{\max}$$

Equation S3

Consequently, if a drug is present and acting at $V_{\max}$ over the time period zero to t then Equation 2 of the main text becomes

$$P_t = P_0 e^{(a-V_{\max})t}$$

Equation S4

where P is number of parasites so that P_0 is the number of parasites at time of treatment and P_t is the number of parasites at time t after treatment. If the drug is not present then $V_{\max} = 0$ and Equation S4 becomes

$$P_t = P_0 e^{at}$$

Equation S5

This assumption will later be relaxed (see Section 4).

(i) 'Hypothetical drug 1' (long half-lives and no stage specificity)

The $V_{\max}$ for the continuous-time methodology is estimated from the PRR_{48} (8). Re-arranging Equation S4 gives

$$\frac{P_0}{P_{48}} = \mathrm{PRR}_{48} = \frac{1}{e^{(a-V_{\max})\times 48}} = e^{-(a-V_{\max})\times 48} = e^{(V_{\max}-a)\times 48}$$

Equation S6

so that

$$V_{\max} = \frac{\ln(\mathrm{PRR}_{48})}{48} + a$$

Equation S7

There is no stage specificity so the same $V_{\max}$ is used in the discrete-time methods, i.e.

$$V'_{\max} = V_{\max} = \frac{\ln(\mathrm{PRR}_{48})}{48} + a$$

Equation S8

(ii) 'Partner drugs' (long half-lives with stage specificity)

The continuous-time method makes no allowance for stage specificity so an 'average' $V_{\max}$ (hereafter referred to as $\hat{V}_{\max}$) is calculated as above (Equation S7), i.e.

$$\hat{V}_{\max} = \frac{\ln(\mathrm{PRR}_{48})}{48} + a$$

Equation S9

The discreet-time models require that the kill rate in the sensitive bins are increased to ensure the same amount of drug killing occurs in the parasites' 48-hour cycle. For the sensitive age-bins

$$\hat{V}'_{\max} = \hat{V}_{\max} \frac{48}{q}$$

Equation S10

where q is the number of age-bins in which killing occurs.

(iii) 'Hypothetical drug 2' (short half-lives and no stage specificity)

Parameterising the continuous-time model must recognise that the drug is not present for the whole 48 hours of the parasite red blood cell (RBC) cycle. The kill rate when the drug is present (denoted $\tilde{V}_{\max}$) must therefore be increased as discussed by Kay and Hastings (8)). We use the symbol t_a to represent the time (in hours) post treatment that the drug is present and killing at maximal rate. The default value used here is 6 hours (3). The principle underlying the calculation is shown graphically on Figure S2. The number of parasites after t_a hours is

$$P_{t_a} = P_0 e^{(a - \tilde{V}_{\max}) t_a}$$

Equation S11

The number of parasites after 48 hours is therefore

$$P_{48} = P_{t_a} e^{a(48 - t_a)}$$

Equation S12

Combining Equation S11 and Equation S12 gives

$$P_{48} = P_0 e^{(a - \tilde{V}_{\max}) t_a} e^{a(48 - t_a)}$$

Equation S13

We can use Equation S13 to obtain $\tilde{V}_{\max}$ as follows. Collect the exponential terms to obtain

$$\frac{P_{48}}{P_0} = e^{(48a - \tilde{V}_{\max} t_a)}$$

Equation S14

Inverting both sides of the equation and taking logs gives

$$\ln\left(\frac{P_0}{P_{48}}\right) = \ln(\mathrm{PRR}_{48}) = \tilde{V}_{\max} t_a - 48a$$

Equation S15

Solving for $\tilde{V}_{max}$ and using Equation S2 to substitute a gives

$$\tilde{V}_{\max} = \frac{\ln(\mathrm{PRR}_{48}) + 48a}{t_a} = \frac{\ln(\mathrm{PRR}_{48}) + \ln(\mathrm{PMR})}{t_a}$$

Equation S16

There is no stage-specific killing so the same value of $\tilde{V}_{max}$ is used in the discrete-time model, i.e.

$$\tilde{V}'_{\max} = \tilde{V}_{\max} = \frac{\ln(\mathrm{PRR}_{48}) + 48a}{t_a}$$

Equation S17

(iv) 'Artemisinin derivatives' (short half-lives and stage specificity)

This type of drug can be calibrated against PRR using the results from the short half-life drug above (Equation S16), i.e.

$$\hat{\tilde{V}}_{\max,48} = \frac{\ln(\mathrm{PRR}_{48}) + 48a}{t_a}$$

Equation S18

For stage specificity, the value of $\hat{\tilde{V}}_{\max,48}$ has to be increased in those age-bin that are sensitive to the drug (as in Equation S10) to give

$$\hat{\tilde{V}}'_{\max,48} = \hat{\tilde{V}}_{\max,48} \times \frac{48}{q}$$

Equation S19

Unfortunately, this produced a rather poor match between the continuous- and discrete-time approaches (see Figure 3 in the main text, particularly for PD4) so an alternative strategy had to be developed as described below.

3. Simulations of artemisinin treatment

Calibration against PRR_{48} did not work for artemisinin drugs because the initial age-bin distribution at time of treatment has such a large effect (Figure 3 in the main text). Artemisinins are given routinely at time 0, 24 and 48 hours (with intermediate dosing in the case of six 12-hourly doses of artemether-lumefantrine, AM-LF). Consequently those infections which are primarily in sensitive age-bins at time of treatment and 48 hour later will be killed by the drug to a much higher extent than those infections which are primarily in drug-insensitive age-bins at time 0 and 48. This effect is "averaged" out in the other three drug types either because they have a long half-live so that parasites have to go through all age-bins during the 48-hour cycle as in 'Hypothetical drug 1' and 'Partner drug', or because they do not have stage specific killing in the case of 'Hypothetical drug 2'. The consequence is that initial age-bins at time of treatment have to be taken into account when simulating treatment with artemisinin-type drugs. This process will be described hereafter.

Simulating individual patients treated with artemisinin

We define the parasite age-bin distribution at time of treatment of the patient, the artemisinin pharmacodynamic profile (the iso- or hyper-sensitive profile; see Section 1) and a PRR_{48} to be achieve (typically 10^4 (5)). We then identify a value of $\hat{\tilde{V}}'_{\max,48}$ that gives the required PRR_{48} for that patient. We could not find an algebraic way of achieving this so $\hat{\tilde{V}}'_{\max,48}$ was obtained by iterating its value using Equation S19 and Equations 4 of the main text to obtain the required PRR_{48}. A problem with using PRR_{48} as the census period for calibration is that it does not capture the effects of subsequent doses of artemisinins; these subsequent doses are not independent of those in the first 24 hours. For example, a dose at 48 hours will target exactly those age-bins already affected by the initial dose at ties zero. It is therefore necessary to define a new census period that occurs after all artemisinin doses have been given. We chose the PRR across two parasite cycles (i.e. at time 2 × 48 = 96 hours) as this census period and it will be denoted PRR_{96}. Note that PRR_{96} is rarely, if ever, reported because limits of

detection inherent in light microscopy make it extremely difficult to quantify the low parasitaemia expected 96 hours after treatment. It is essentially a theoretical metric used for the computations described below. To summarise the process: A value of $\hat{\tilde{V}}'_{\max,48}$ is obtained by iteration to get the required PRR_{48}, using Equation S19 and Equations 4 of the main text, i.e. assuming a given age-bin distribution of parasites at time of treatment and treatment at times 0, 24 and 48 hours. Once this value of $\hat{\tilde{V}}'_{\max,48}$ has been obtained by iteration, the simulation is then continued on from 48 hours to 96 hours to obtain the PRR_{96}. This value of PRR_{96} allows a continuous-time equivalent for the artemisinin kill rate in that patient $\hat{\tilde{V}}'_{\max,96}$ to be calculated using an analogous method to the one used to derive Equation S16 (and shown in Figure S2) that accounts for the short half-life of the drug, its stage-specificity and for multiple dosing. The method is as follows. We initially assume that three doses are given at times 0, 24 and 48 hours. The number of surviving parasites after 24 hours (i.e. immediately before the second dose) is

$$P_{24} = P_0 e^{(a-\hat{\tilde{V}}_{\max,96})t_a} e^{a(24-t_a)}$$

Equation S20

The number of surviving parasites after 48 hours, immediately before the third dose is

$$P_{48} = P_{24} e^{(a-\hat{\tilde{V}}_{\max,96})t_a} e^{a(24-t_a)}$$

Equation S21

And the number of surviving parasites at 96 hours, i.e. over two RBC life-cycles, is

$$P_{96} = P_{48} e^{(a-\hat{\tilde{V}}_{\max,96})t_a} e^{a(48-t_a)}$$

Equation S22

Substituting P_{24} and P_{48} into Equation S22 gives

$$P_{96} = P_0 e^{\left[(a-\hat{\tilde{V}}_{\max,96})t_a\right]^3} e^{[a(24-t_a)]^2} e^{a(48-t_a)}$$

Equation S23

The reduction in parasite number over two RBC life-cycles, i.e. PRR_{96}, can be calculated by using the rule that $(m^x)^y=m^{xy}$, collecting the terms in the exponents, and moving P_0 to the left-hand side

$$\frac{P_{96}}{P_0} = \mathrm{PRR}_{96} = e^{(96a-3t_a\hat{\tilde{V}}_{\max,96})}$$

Equation S24

This makes intuitive sense as it states that the number of parasites 96 hours after start of treatment, scaled by initial number, is the amount of growth that has occurred over that 96 hours (i.e. 96a in the exponent) discounted by the magnitude ($\hat{\tilde{V}}'_{\max,96}$) and duration ($3t_a$) of

drug killing. It is therefore easy to extend this to other regimens, e.g. if AM-LF is given six times during a regimen then the amount of drug killing becomes $6t_a$.

Taking reciprocals of both sides of Equation S24 and transforming to natural logarithms gives

$$\ln\left(\frac{P_0}{P_{96}}\right) = \ln(\mathrm{PRR}_{96}) = 3t_a\hat{\tilde{V}}_{\max,96} - 96a$$

Equation S25

Re-arranging and substituting a (Equation S2) gives

$$\hat{\tilde{V}}_{\max,96} = \frac{\ln(\mathrm{PRR}_{96}) + 96a}{3t_a} = \frac{\ln(\mathrm{PRR}_{96}) + 2\ln(\mathrm{PMR})}{3t_a}$$

Equation S26

This equation is analogous to Equation S16 and enables us to get a continuous-time approximation $\hat{\tilde{V}}'_{\max,96}$ for the PRR_{96} simulated for any given patient. The equivalence of the discrete and continuous-time approaches is demonstrated in Figure 4 of the main text.

Simulating population-wide use of artemisinins

It would be possible to simulate a large patient population using the discrete-time approach. However we want a computational shortcut using the much faster continuous time methodology. This section shows the methodology we used to achieve this.

An appropriate value of $\hat{\tilde{V}}'_{\max,48}$ for any individual patient can be obtained by iteration to obtained the required PRR_{48} (and hence PRR_{96}) for that patient and obtained using Equation S26 as described above. The next problem is to obtain the distribution of $\hat{\tilde{V}}'_{\max,96}$ in the patient population. The value for any real patient is composed of three elements

$$\hat{\tilde{V}}_{\max,96} = \hat{\tilde{\mu}}_{\max} + e(n) + e(s)$$

Equation S27

where $\hat{\tilde{\mu}}_{\max}$ is the mean value of $\hat{\tilde{V}}'_{\max,96}$ in the parasite population, $e(n)$ is the effect of natural variation in parasite drug sensitivity and $e(s)$ is the effect of infections' age-bin distribution at time of treatment. Equation S27 reveal a strategy for mass simulation of artemisinin treatment. It is first necessary to obtain an estimate for $\hat{\tilde{\mu}}_{\max}$, then set $e(n) = 0$ and simulate a whole patient population. The resulting variation in $\hat{\tilde{V}}'_{\max,96}$ will equal the variation in $e(s)$. Natural variation is generally assumed to have a coefficient of variation (CV) of around 0.3

(8, 9) so we will have estimates of all three component of Equation S27 which will allow simulation of whole patient populations.

Estimating the mean value of $\hat{\tilde{V}}'_{\max,96}$ (i.e. $\hat{\tilde{\mu}}_{\max}$)

The first problem is to decide a typical patient age-bin distribution to obtain the 'mean' population value of $\hat{\tilde{V}}'_{\max,96}$, i.e. $\hat{\tilde{\mu}}_{\max}$, in Equation S27. Table S1 showed that total kill rate depends on the age-bin distribution at time of treatment so it is necessary to identify a 'typical' value to obtain the mean artemisinin kill rate. The results presented here were calibrated according to the assumption that the parasites are completely asynchronous at the time of treatment, i.e. uniformly distributed across age-bins, that parasites have the iso-sensitive pharmacodynamic profile with gradual sequestration between age-bins 11 and 14 as described above. This gave a value of $\hat{\tilde{V}}'_{\max,96}$ = 1.164. Using the approach described above to convert discrete-time calibration $\hat{\tilde{V}}'_{\max}$ to continuous-time (i.e. running simulations out to 96 hours and using Equation S26 to obtain $\hat{\tilde{V}}'_{\max,96}$) gave a mean value of $\hat{\tilde{V}}'_{\max,96}$, i.e. $\hat{\tilde{\mu}}_{\max}$ = 0.524 (Table 1 of main text). This is regarded as the mean value in the populations and was the value used to produce Figure 4 in the main text and Equation S4 onwards in this supplemental material.

Our intention is to develop a methodology rather than coercing people to follow a specific calibration. We have described the methodology in detail so that users can substitute their own preferred pharmacokinetics, pharmacodynamics and age-bin distribution (*cf.* Table S1).

Estimating the impact of the age-bin distribution of parasites at time of treatment, i.e. *e*(*s*)

The likely impact of age-bin distribution of parasites on artemisinin drug effectiveness was investigated. The artemisinin pharamcodynamic profile was held constant (i.e. iso-sensitive profile; Figure 1), $\hat{\tilde{V}}'_{\max,96}$ was held at 1.164, while the mean of parasite age-bin distributions at time of treatment were varied. It was assumed all distributions were normally distributed with SD = 5 hours. Figure S1 illustrates how varying age-bin distribution of time of treatment affects artemisinin killing rates (PRR values) and also the continuous-time kill rate. The PRR_{96} varies substantially and $\hat{\tilde{V}}'_{\max,96}$ varies almost two-fold in this example, i.e. from ~0.5 to ~0.9 depending on the mean age-bin of the infection.

The methods above describe how $\hat{\tilde{V}}'_{\max,96}$ can be calculated by iteration for one patient. The essence of the methodology is now to repeat these calculations for a large simulated patient populations (SPPs) to quantify how the differences in patients' age-bin distributions at time of treatment creating variation around the mean value of $\hat{\tilde{\mu}}_{\max}$ = 0.524 obtained as described

above. The critical point is that $\hat{\tilde{V}}'_{\max,96}$ = 1.164 will be held constant in each patient. The discrete-time model is then run for 96 hours to obtain each patient's PRR_{96} which can then be used by Equation S26 to calculate $\hat{\tilde{V}}'_{\max,96}$ for each patient. The resulting distribution of $\hat{\tilde{V}}'_{\max,96}$ is shown in Figure S7. The variation in $\hat{\tilde{V}}'_{\max,96}$ that is likely to occur in patient populations treated by artemisinins in different epidemiological/clinical settings (as reflected in our simulated patient populations, SPP, see above). When infections have low levels of synchronicity at time of treatment (i.e. SPP1 with 30% of patients have asynchronous infections, while the remaining 70% have SD from 6–8 hours) then variance introduced by differences in patients differing age-bin distributions at time of treatment, *e*(*s*), is likely to be relatively low. If patients present for treatment early in their infection cycle such that their infections are relatively synchronised (SPP2 and SPP3, i.e. SD from 2–4 hours) then the variation in *e*(*s*) is substantially increased. Interestingly, it appears that synchronicity is the main source of variation as patients presenting with parasites mainly in early bins (SPP2) show little difference in variation in $\hat{\tilde{V}}'_{\max,96}$ compared to those presenting with their infections in a wider range of mean ages (SPP3). Figure S7 suggests that *e*(*s*), variation in $\hat{\tilde{V}}'_{\max,96}$ caused by differing age-bin distributions of infections at time of treatment, is from ~0.4 to ~0.8 which is a difference of 0.4 in absolute units or roughly a two-fold range. A key question is therefore whether variation due to initial bin distribution scales with $\hat{\tilde{\mu}}_{\max}$ or remains constant, e.g. if $\hat{\tilde{\mu}}_{\max}$ is doubled, does the variation remain at 0.4 units around the new value 2 × $\hat{\tilde{\mu}}_{\max}$, or does this interval increase to 0.8 so that the range is again about two-fold? Similarly, if $\hat{\tilde{\mu}}_{\max}$ is halved, does the distribution remain at 0.4 or does it narrow to 0.2 maintaining a two-fold variation around $\hat{\tilde{\mu}}_{\max}$ /2? Figure S8 and Figure S9 suggest that variation around $\hat{\tilde{V}}'_{\max,96}$ does scale to maintain a two-fold variation. Elementary algebra show that the limits of this parameter interval (PI) that give a two-fold variation around any given value of $\hat{\tilde{V}}'_{\max,96}$ are

$$\mathrm{PI} = \hat{\tilde{V}}_{\max} \pm \frac{\hat{\tilde{V}}_{\max,96}}{3}$$

Equation S28

In other word *e*(*s*) in Equation S27 will follow a uniform distribution between the limits given in Equation S28.

Incorporating stage specificity into mass simulations

Three factors affecting artemisinin treatment effectiveness need to be incorporated into simulations of patient populations. Firstly, natural variation in human pharmacokinetics such as drug elimination rate and volume of distribution which determine how long and at what concentration the drug resides in the patient's body. Secondly, the natural variation in the

parasite pharmacodynamic parameters such as maximal kill rate and the concentration at which 50% of maximal killing occurs (IC_{50}) which determine the degree of drug sensitivity of a parasite population. These first two factors are incorporated in mass simulations using continuous-time models by allowing random variation around the mean values. For example, in our previous simulations we allowed a CV of 0.3 around parameter values. The third source of variation in artemisinin killing is the distribution of the parasites among drug-sensitive and drug-insensitive age-bins at the time of treatment. This additional variance due to age-bin distribution at time of treatment can be incorporated by allowing an additional two-fold variation in $\tilde{\hat{V}}'_{\max,96}$ values (Equation S28).

The variance in $\tilde{\hat{V}}'_{\max,96}$ due to bin distribution, $e(s)$ can be added to the natural variation $e(n)$ around $\tilde{\hat{\mu}}_{\max}$ in the continuous-time simulation for the defined patient population (Equation S27).

The final step in the methodological design pathway is now to confirm that stage specificity may be easily incorporated in mass simulations in practice, and that no unanticipated problems arise. Another consideration is to examine the likely impact of incorporating stage specificity on previous results. We address both issues by incorporating these computational shortcuts and repeating our mass simulations of artesunate-mefloquine (AS-MQ) and AM-LF treatment described previously (8). The failure rates to both drugs are shown on Figure 2 of that paper; there seemed little point in using the basal, default parameters (see Table S1 of (8)) as these resulted in very low failure rates so we selected parameter combination that gave a 10–20% failure rate in order to make sure we would capture differences in cure rates due to the incorporation of stage specificity. Simulation of AS-MQ used an IC_{50} for MQ that was increased 25-fold above its default value while IC_{50} for both, AS and DHA, were kept at their original default value. These parameters gave a failure rate of 18% (Panel E, Figure 2 of (8)). Simulation of AM-LF used a LF IC_{50} that was increased 50-fold above its default value while IC_{50} for both, AM and DHA, were increased 20-fold above their original default value. These parameters gave failure rate of 11% (Panel F, Figure 2 of (8)).

Mass simulations of 10,000 patients were run using the same seed value. Each patient was tracked for 100 days following treatment with parameter values and their associated variation as described in Table S1 of (8), except for the increased IC_{50} values described above.

The effects of the artemisinins' stage specificity were incorporated by increasing the variation associated with their maximum kill rate $\tilde{\hat{V}}'_{\max,96}$. This was a two-part process in which the $\tilde{\hat{V}}'_{\max,96}$ of each infection was initially sampled from the 'natural' distribution (originally described in (8)) and then re-sampled from a uniform distribution with a two-fold range around that initial $\tilde{\hat{V}}'_{\max,96}$ value. The first stage incorporates the variability observed within patients' infections while the second stage incorporates the variability in kill rates introduced by variation in parasite bin distribution at time of treatment (Equation S28).

Above we argue that $\tilde{\hat{V}}'_{\max,96}$ for the artemisinins should be calibrated using PRR_{96} (in contrast to our previous work which calibrated the kill rate ($V_{\max}$) using PRR_{48} (8)). In order to

account for the effects of recalibrating the artemisinins' kill rate we reduced mean $\hat{\tilde{V}}'_{\max,96}$ by a factor of 2, i.e. from 1.15 per hour (27.6 per day) to 0.6 per hour (14.4 per day). This is further explained in the Discussion of the main text.

The stage specificity of LF and MQ are less consequential because of their long half-lives (see later) and mainly affect the predicted minimum number of parasites following treatment. The methodology assumes that an infection is cured if this predicted number falls below 1. We maintain this criterion but now record the minimum predicted number of parasites in each patient to check how close it lies to this critical value of 1.

4. Running discrete- and continuous-time simulation in practice: relaxing the simple drug present/absent assumption

The calibrations made above rest on the assumption that drug(s) are either present and killing at their maximal effect or are absent (or, more correctly, present at concentrations sufficiently low that they have no effects on the parasite). This was done to make the computational approaches more transparent. In practice, investigators will almost certainly wish to apply a more nuanced pharmacokinetic/pharmacodynamic approach that can be brought in as follows. The method is based on Equation 2 of the main text which gives the number of parasites *P* at time *t* after treatment (P_t), i.e.

$$P_t = P_0 e^{at} e^{-\int_0^t f(C)dt}$$

Equation S29

which uses drug concentration at time *t* to obtain a kill rate by a Michaelis-Menton equation as in Equation 3 of the main text, i.e.

$$f(C) = V_{\max}\left(\frac{(C_t)^n}{(C_t)^n + \mathrm{IC}_{50}{}^n}\right)$$

Equation S30

where V_{max} has been calculated according to drug type (Table 1 of the main text). The simple approach used in the main text assumes drug is either present at sufficient concentrations to kill at V_{max}, or is so low as to have no killing effect (represented by or $Z_t = 1$ or $Z_t = 0$ respectively in Equation 4). In reality, C_t, the drug concentration at time *t*, may take a few hours to reach maximal concentrations as absorption and drug conversion processes take place and decays gradually over time violating this assumption of a strict drug presence/absence.

The construction of *f*(*C*) therefore requires a function describing drug concentration over time following treatment, which is then converted to a drug kill rate. In this illustrative example we use the simplest pharmacokinetic equation (used previously by several authors e.g. (9-13)) describing drug concentration as a function of time following a drug dose that instantaneously distributes into a single physiological compartment, so that

$$C_t = \frac{D}{Vd} e^{-kt}$$

Equation S31

where C_t is the drug concentration [mg/L] at time t after treatment, D is the dose [mg], Vd is the volume of distribution [L], and k is the drug elimination rate per hour or per day. Variants of Equation S31 that allow for multiple drug dosing and physiological processes such as drug absorption across the gut, conversion to active metabolites, and distribution into different physiological compartments are discussed elsewhere (8).

The continuous-time methodology simply uses Equation S29 to describe the whole treatment dynamics. The use of a single differential equation (i.e. Equation S29) is more elegant and convenient as it makes it unnecessary to stop and restart the simulations at the boundaries where drugs change from present to absent (*cf*. Figure S2) because the boundaries are replaced by the gradual, time-dependent decay of drug concentration described by Equation S31.

The discrete-time analyses require the calculation of the proportion of parasites in age-bin b surviving a one hour period i.e. $\Psi^{b,t}$ as in Equation 5 of the main text and as subsequently used in Equations 6 and 7 of the main text). This can be calculated from the above pharmacological model as follows. First incorporate stage specific killing into Equation S30 (denoted by the prime symbol) as

$$f(C') = Y_b f(C)$$

Equation S32

then re-arrange Equation S29 to obtain

$$\Psi^{b,t} = \frac{P_{t+1}}{P_t} = e^{at} e^{-\int_t^{t+1} f(C')dt}$$

Equation S33

There is no continuous growth in the discrete-time method because parasite reproduction is incorporated after parasites reach 48 hours (Equation 7 of the main text) so $a = 0$ and

$$\Psi^{b,t} = e^{-\int_t^{t+1} f(C')dt}$$

Equation S34

This can then be used in the discrete-time method as specified in Equations 6 and 7 in the main text.

References

1. **Saralamba S, Pan-Ngum W, Maude RJ, Lee SJ, Tarning J, Lindegardh N, Chotivanich K, Nosten F, Day NP, Socheat D, White NJ, Dondorp AM, White LJ.** 2011. Intrahost modeling of artemisinin resistance in *Plasmodium falciparum*. Proc Natl Acad Sci U S A **108:**397-402.
2. **White NJ.** 2011. The parasite clearance curve. Malar J **10:**278.
3. **Zaloumis S, Humberstone A, Charman SA, Price RN, Moehrle J, Gamo-Benito J, McCaw J, Jamsen KM, Smith K, Simpson JA.** 2012. Assessing the utility of an anti-malarial pharmacokinetic-pharmacodynamic model for aiding drug clinical development. Malar J **11:**303.
4. **White NJ, Krishna S.** 1989. Treatment of malaria: some considerations and limitations of the current methods of assessment. Trans R Soc Trop Med Hyg **83:**767-777.
5. **White NJ.** 1997. Assessment of the pharmacodynamic properties of antimalarial drugs *in vivo*. Antimicrobial Agents and Chemotherapy **41:**1413-1422.
6. **Sanz LM, Crespo B, De-Cózar C, Ding XC, Llergo JL, Burrows JN, García-Bustos JF, Gamo F-J.** 2012. *P. falciparum In Vitro* Killing Rates Allow to Discriminate between Different Antimalarial Mode-of-Action. PLoS ONE **7:**e30949.
7. **Klonis N, Xie SC, McCaw JM, Crespo-Ortiz MP, Zaloumis SG, Simpson JA, Tilley L.** 2013. Altered temporal response of malaria parasites determines differential sensitivity to artemisinin. Proceedings of the National Academy of Sciences doi:10.1073/pnas.1217452110.
8. **Kay K, Hastings IM.** 2013. Improving pharmacokinetic-pharmacodynamic modeling to investigate anti-infective chemotherapy with application to the current generation of antimalarial drugs. PLoS Comput Biol **9:**e1003151.
9. **Winter K, Hastings IM.** 2011. Development, evaluation, and application of an *in silico* model for antimalarial drug treatment and failure. Antimicrob Agents Chemother **55:**3380-3392.
10. **Austin DJ, White NJ, Anderson RM.** 1998. The dynamics of drug action on the within-host population growth of infectious agents: melding pharmacokinetics with pathogen population dynamics. J Theor Biol **194:**313-339.
11. **Hoshen MB, Stein WD, Ginsburg H.** 1998. Modelling the chloroquine chemotherapy of falciparum malaria: the value of spacing a split dose. Parasitology **116:**407-416.
12. **Hoshen MB, Stein WD, Ginsburg HD.** 2001. Pharmacokinetic-pharmacodynamic modelling of the antimalarial activity of mefloquine. Parasitology **123:**337-346.
13. **Simpson JA, Agbenyega T, Barnes KI, Di Perri G, Folb P, Gomes M, Krishna S, Krudsood S, Looareesuwan S, Mansor S, McIlleron H, Miller R, Molyneux M, Mwenechanya J, Navaratnam V, Nosten F, Olliaro P, Pang L, Ribeiro I, Tembo M, van Vugt M, Ward S, Weerasuriya K, Win K, White NJ.** 2006. Population pharmacokinetics of artesunate and dihydroartemisinin following intra-rectal dosing of artesunate in malaria patients. PLoS Med **3:**e444.

Table S1. Calibration of artemisinin drug kill rates. The pharmacodynamic profiles of artemisinins are the 'iso-sensitive' or the 'hyper-sensitive' profiles as illustrated on Figure 1 of the main text. PRR_{48} is parasite reduction ratio after 48 hours (assumed to be ~10^4). The prefix 't' indicates 'true' PRR_{48}, i.e. calculated assuming that all parasites are detected, while the prefix 'a' indicates that PRR_{48} calculations use only the 'apparent' or 'observable' non-sequestered parasites. In the latter case sequestration may be immediate ('imm'; all parasites immediately disappear after age-bin 14) or more gradual ('grad'; parasites start to gradually sequester after age 11 hours as described previously (1)). We obtained the artemisinin kill rate, $\hat{\tilde{V}}'_{\max,48}$, by iteration to produce a PRR_{48} of ~10^4 with a precision of 0.1% (hence the PRR_{48} is never exactly 10^4 in the Table). The value of $\hat{\tilde{V}}'_{\max,48}$ was then used to calculate PRR_{96} and $\hat{\tilde{V}}'_{\max,96}$ as described in Section 3.

**Pharmacodynamic profile**	**Bin distribution at start of treatment (mean, SD)**	**Calibration**	**PRR_{48}**	$\hat{\tilde{V}}'_{\max,48}$
Iso-sensitive	uniform	$tPRR_{48}$	10,037	1.637
Iso-sensitive	uniform	$aPRR_{48}$ (imm)	10,054	1.164
Iso-sensitive	uniform	$aPRR_{48}$ (grad)	9,984	1.364
Iso-sensitive	normal (10.5, 5)	$tPRR_{48}$	10,057	1.415
Iso-sensitive	normal (10.5, 5)	$aPRR_{48}$ (imm)	9,945	1.087
Iso-sensitive	normal (10.5, 5)	$aPRR_{48}$ (grad)	9,933	1.143
Hyper-sensitive	uniform	$tPRR_{48}$	10,017	1.711
Hyper-sensitive	uniform	$aPRR_{48}$ (imm)	9,993	1.780
Hyper-sensitive	uniform	$aPRR_{48}$ (grad)	10,036	1.766
Hyper-sensitive	normal (10.5, 5)	PRR_{48}	9,988	1.776
Hyper-sensitive	normal (10.5, 5)	$aPRR_{48}$ (imm)	9,994	1.808
Hyper-sensitive	normal (10.5, 5)	$aPRR_{48}$ (grad)	9,974	1.804

Table S2. Result of mass simulations of treatment by artemether-lumefantrine (AM-LF) and artesunate-mefloquine (AS-MQ). Simulations of 10,000 individuals were based on the original model of Kay and Hastings (8) with some of the individual drug IC_{50} values changed as described in the main text to generate predicted drug failure rates in the base model (mean $\hat{\tilde{V}}'_{\max,96}$ = 27.6 and no correction for stage specificity) of 8% and 15% for AM-LF and AS-MQ respectively. The value of mean artemisinin kill rate, $\hat{\tilde{V}}'_{\max,96}$, was either 27.6 per day (as in the original paper (8)) or reduced to 14.4 per day (for reasons explained in the main text). The impact of correcting ('corr.') for the effects of parasites age-bin distribution at time of treatment was also investigated (see Equation S28). The minimum parasites number was recorded for each individual to find the percentage of simulations where the minimum number of parasites was close (within two log10 units) to the cure/fail threshold of one, i.e. between 0.01 and 100.

	$\hat{\tilde{V}}'_{\max,96}$		Min. Parasite number (%)						Treatment outcome
**Drug**	**mean**	**corr.**	**<0.01**	**0.01–0.1**	**0.1–1**	**1–10**	**10–100**	**>100**	**% cured**
AM-LF	27.6	No	89.71	1.42	1.16	1.24	1.12	5.35	92.29
AM-LF	27.6	Yes	88.93	1.39	1.44	1.19	0.94	6.11	91.76
AM-LF	14.4	No	76.73	2.66	2.37	2.25	2.50	13.49	81.76
AM-LF	14.4	Yes	75.87	2.70	2.45	2.39	2.33	14.26	81.02
AS-MQ	27.6	No	77.91	3.60	3.23	3.24	3.09	8.93	84.74
AS-MQ	27.6	Yes	77.17	3.57	3.39	2.94	3.36	9.57	84.13
AS-MQ	14.4	No	59.49	2.40	2.64	2.94	3.01	29.52	64.53
AS-MQ	14.4	Yes	59.89	2.39	2.69	2.85	3.13	29.05	64.97

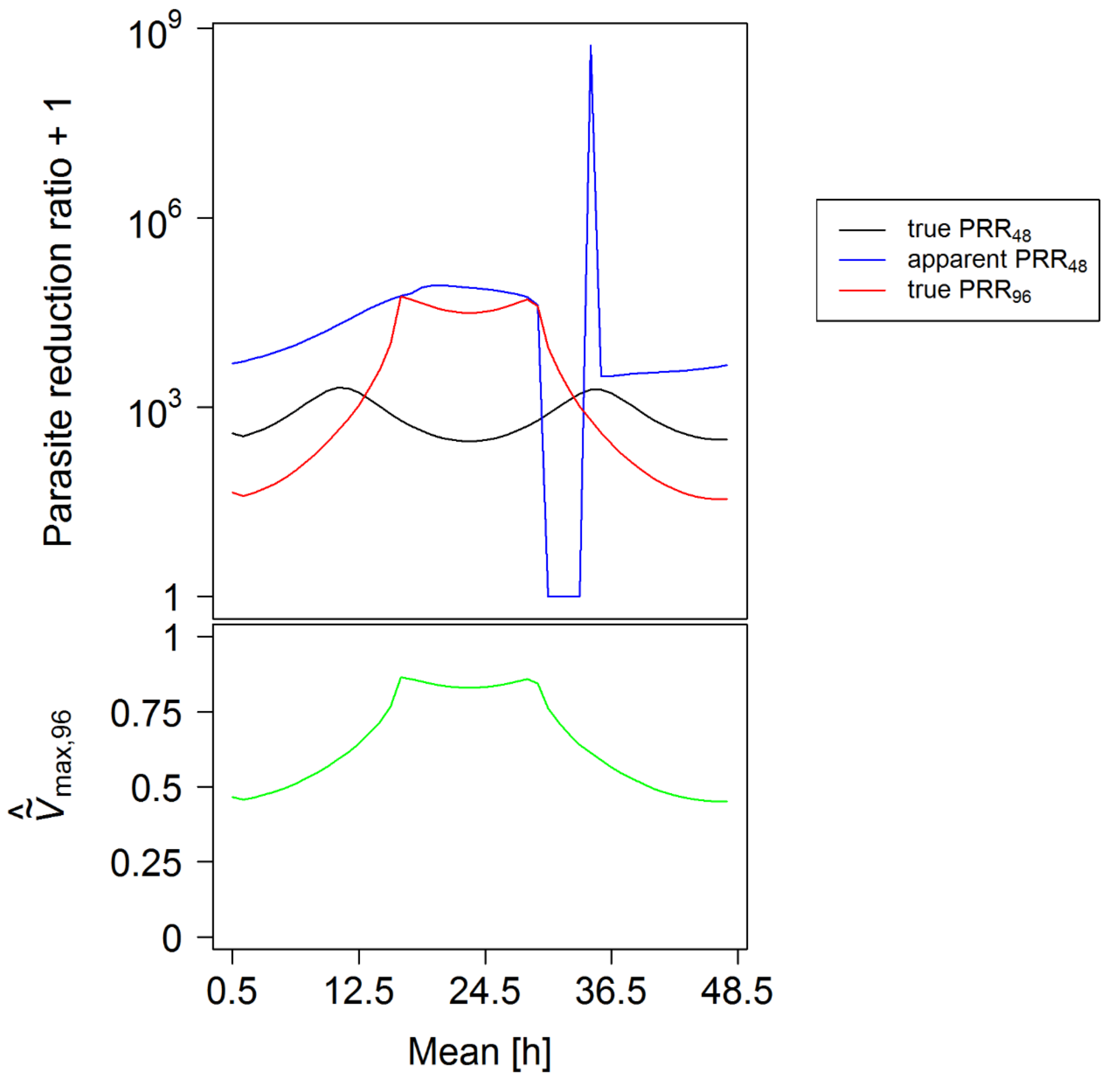

Figure S1. How starting age-bin distributions affect the parasite reduction ratio (PRR) and the effective parasite killing rate ($\hat{\tilde{V}}'_{max,96}$) of artemisnins. Parasite drug sensitivity is described by the iso-sensitive pharamacodynamic profile (Figure 1 in the main text). Age-bins at time of treatmenet are normally distributed (with SD = 5 hours) but have different mean values; the latter are plotted along the x-axis.

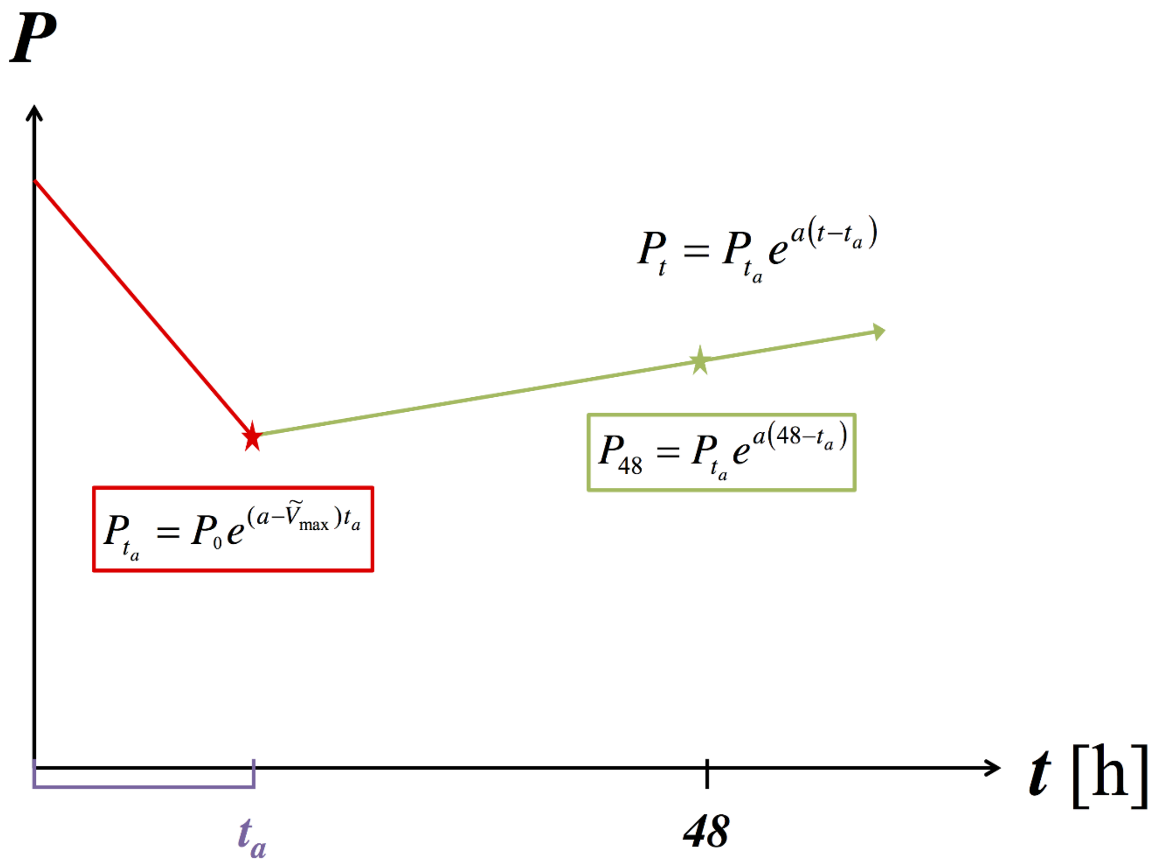

Figure S2. Estimating parasite number after treatment by a short half-life drug and equal killing in all age-bins. Parasite number P is shown on a log scale over time t (in hours) after treatment. The red line shows how the initial number of parasites P_0 drops while the drug is present and acting with maximal kill rate $\tilde{V}_{max}$. At time point t_a the drug concentration has declined such that it can no longer kill parasites (see Section 2). The parasite number at t_a (red star) can be calculated using the equation in the red box (i.e. Equation S11). After time t_a the parasites start to grow (green line) and the number of parasites can be calculated using the equation in the green box (i.e. Equation S12) to obtain the parasite reduction ratio (PRR_{48}, see Equation S6) after 48 hours (green star).

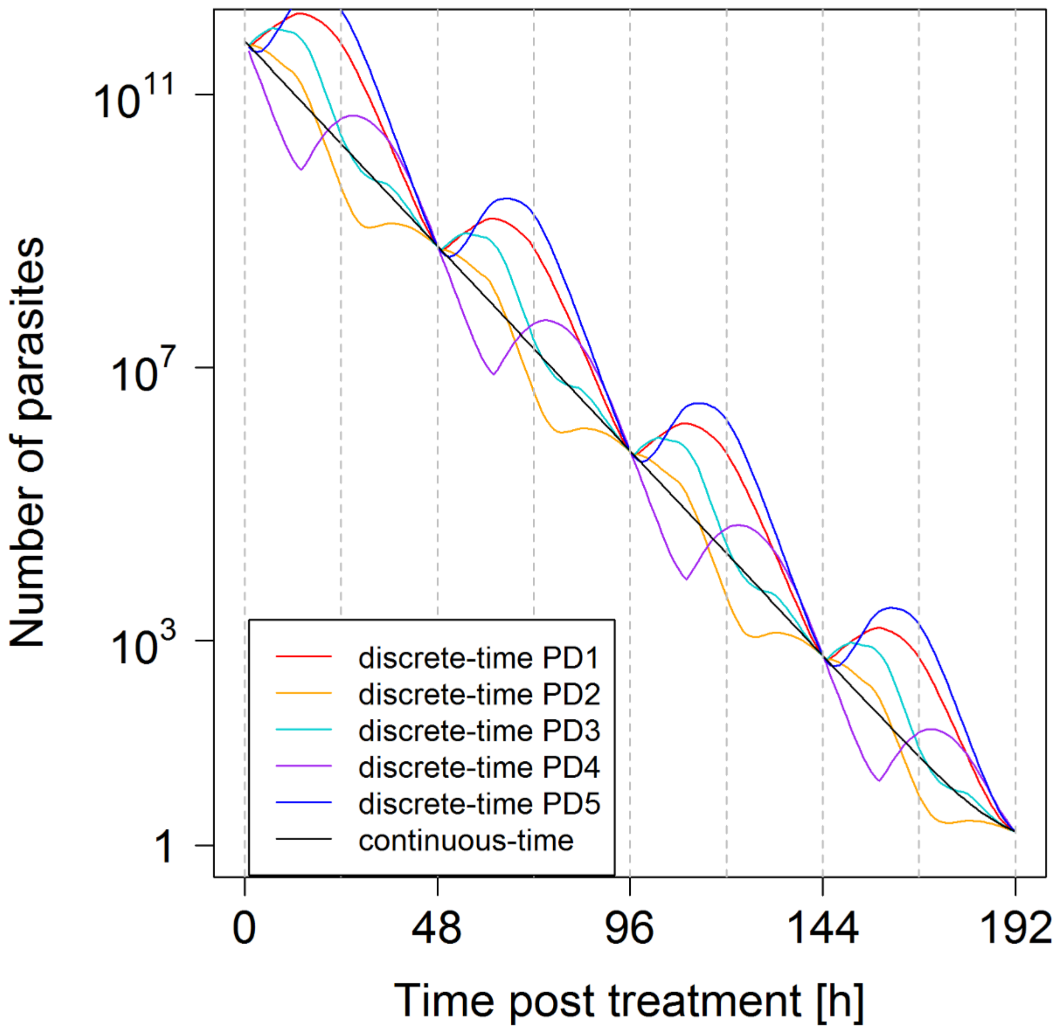

Figure S3. Changes in parasite numbers following treatment by a drug with long half-life and stage specific killing (e.g. piperaquine). This was produced using the pharmacodynamic profile of drug 'piperaquine'. Parasites present at time of treatment were distributed among age-bins according to paradigm distributions (PD) 1–5 described in Section 1. The discrete-time model used drug killing rate $\hat{V}'_{\max}$ = 0.3684, $Y_b = 1$ for age-bins 12 to 36 inclusive and $Y_b = 0$ for age-bins 0 to 11 and 37 to 48 inclusive and the continuous-time model used drug killing rate $\hat{V}_{\max}$ = 0.1919. Note that the number of parasites is the true number, i.e. circulating plus sequestered, plus one (it is conventional to plot parasites + 1 when using a log scale because log(0) is undefined).

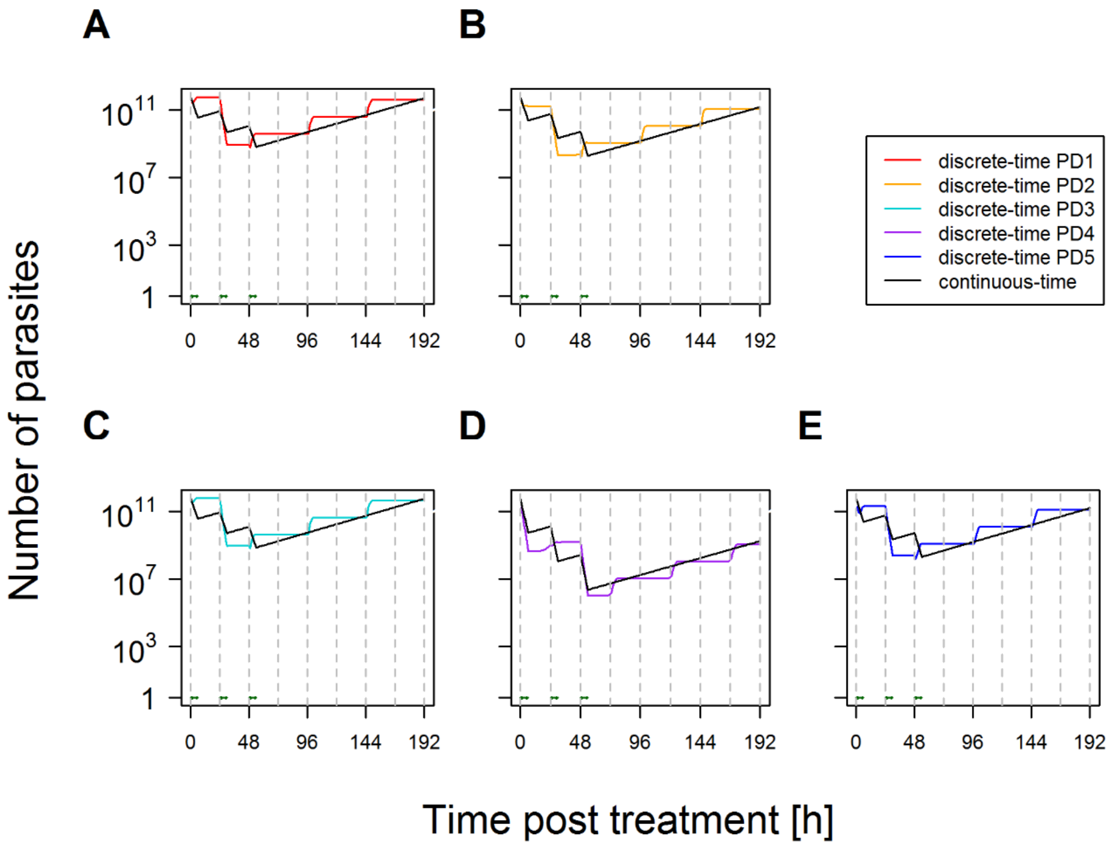

Figure S4. Changes in parasite numbers following treatment by a drug with short half-life and stage specific killing such as artemisinin and assuming no drug is present after96 hours. The difference between discrete- and continuous-time predictions arise because parasites reproduce at the end of their 48-hour cycle. This is tracked in the discrete-time methods but not by its continuous-time equivalent. Calibration (and dynamics up to 96 hours) are as for Figure 4 of the main text. Note that the number of parasites is the true number, i.e. circulating plus sequestered, plus one (it is conventional to plot parasites + 1 when using a log scale because log(0) is undefined).

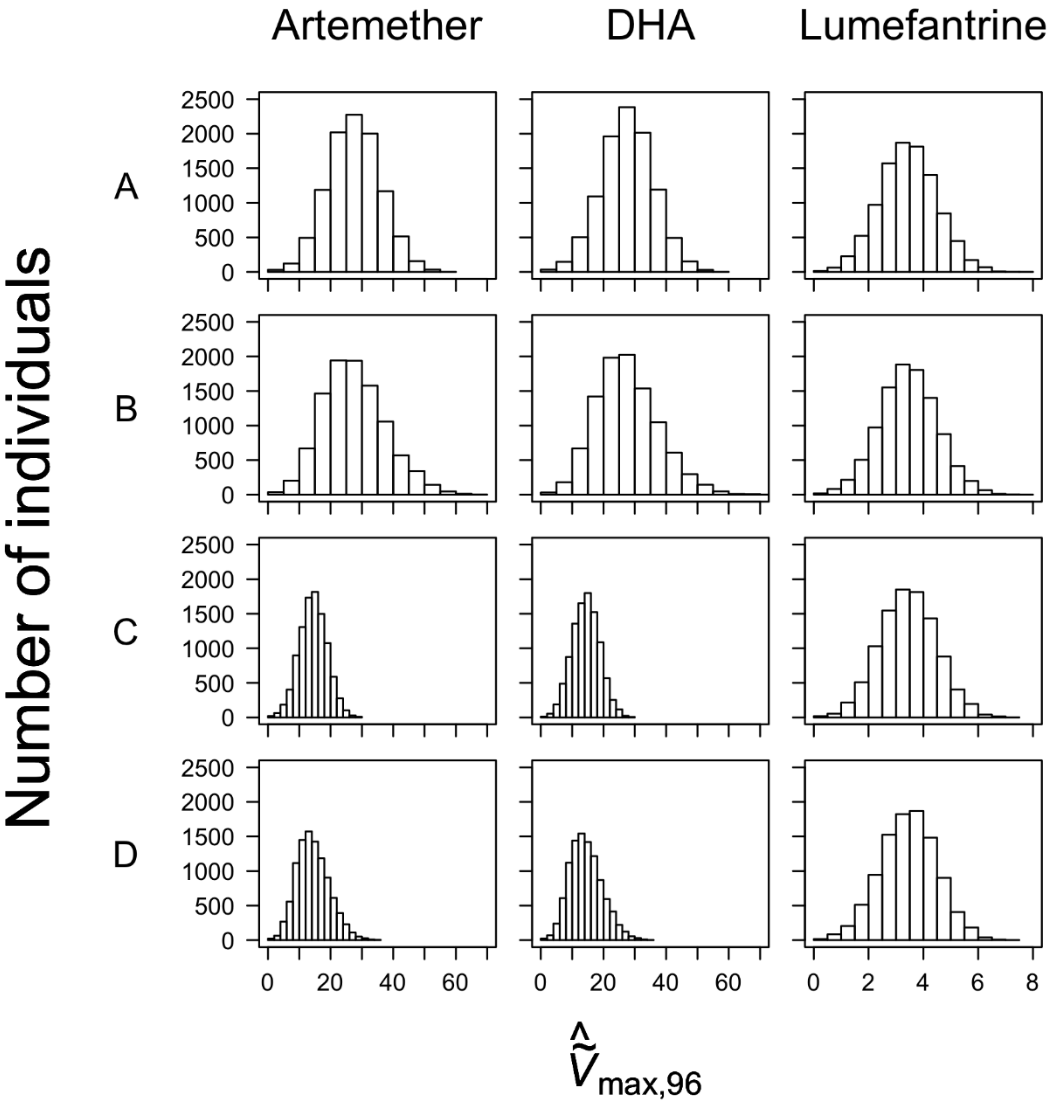

Figure S5. Distribution of artemisinin drug kill rates, $\hat{\tilde{V}}_{max,96}$, among patients in a mass simulations of 10,000 patients treated with artemether-lumefantrine (AM-LF). (A) Original model from Kay and Hastings (8), i.e. the mean drug killing rate of $\hat{\tilde{V}}_{max,96}$ = 27.6 per day for artemether (AM) and its active metabolite dihydroartemisinin (DHA) with no correction for stage specific killing. **(B)** As for (A) but with correction for stage specific killing of artemisinins included by resampling every value chosen from (A) from a two-fold uniform variation around that chosen value (Equation S28). **(C)** Mean drug killing rate $\hat{\tilde{V}}_{max,96}$ for AM and DHA has been reduced to 14.4 with no correction for stage specific killing. **(D)** As for (C) but with correction for stage specific killing of artemisinins included by resampling every value chosen from (C) from a two-fold uniform variation around that chosen value (Equation S28). Note that neither the mean drug killing rate, $\hat{\tilde{V}}_{max,96}$, nor its variance was altered for lumefantrine (LF) and that the third column is included simply to make this point clear and to act as an internal 'control' demonstrating that neither the mean nor variance had changed for LF.

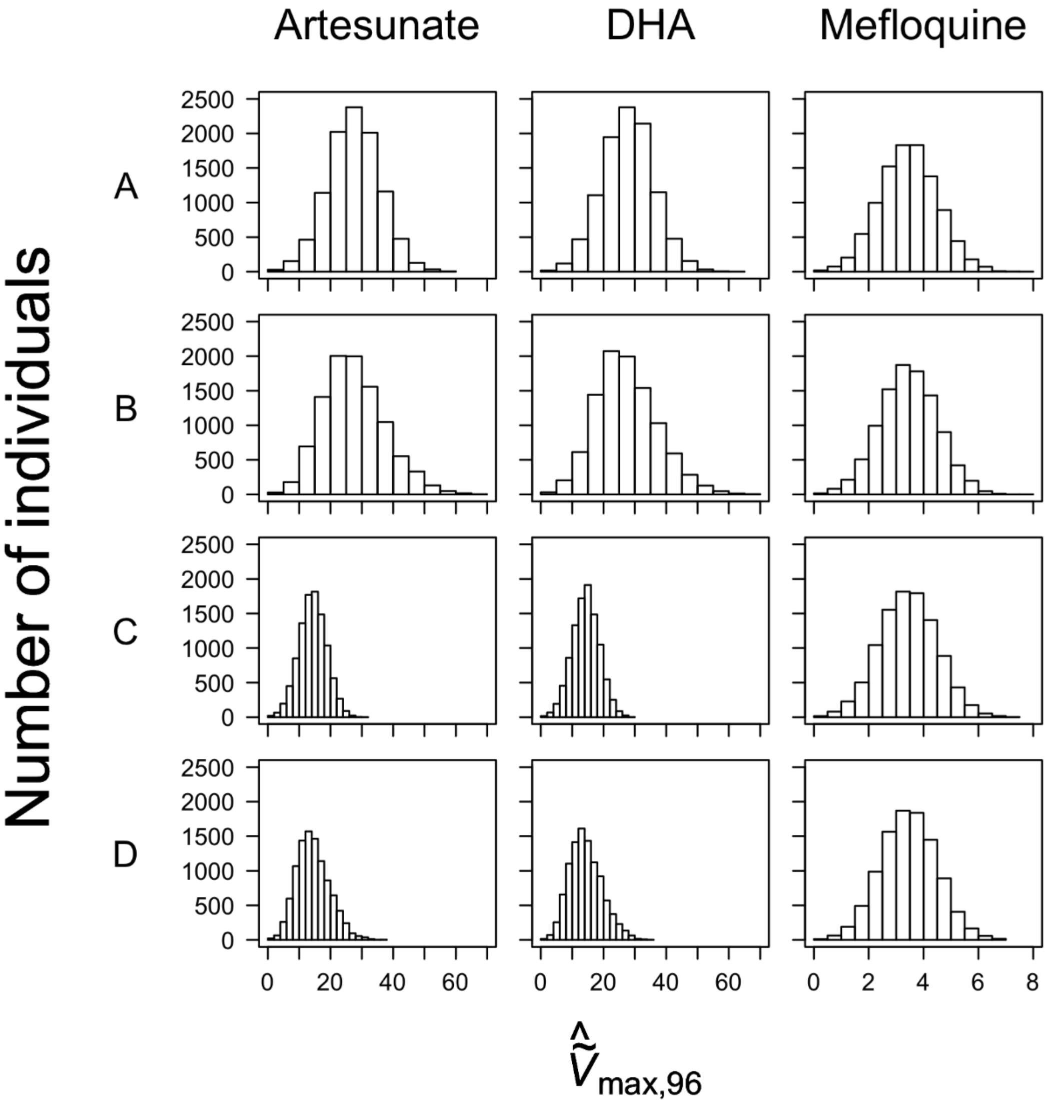

Figure S6. Distribution of artemisinin drug kill rates, $\hat{\tilde{V}}_{max,96}$, among patients in a mass simulations of 10,000 patients treated with artesunate-mefloquine (AS-MQ). (A) Original model from Kay and Hastings (8), i.e. the mean drug killing rate of $\hat{\tilde{V}}_{max,96}$ = 27.6 per day for artesunate (AS) and its active metabolite dihydroartemisinin (DHA) with no correction for stage specific killing. **(B)** As for (A) but with correction for stage specific killing of artemisinins included by resampling every value chosen from (A) from a two-fold uniform variation around that chosen value (Equation S28). **(C)** Mean drug killing rate $\hat{\tilde{V}}_{max,96}$ for AS and DHA has been reduced to 14.4 with no correction for stage specific killing. **(D)** As for (C) but with correction for stage specific killing of artemisinins included by resampling every value chosen from (C) from a two-fold uniform variation around that chosen value (Equation S28). Note that neither the mean drug killing rate, $\hat{\tilde{V}}_{max,96}$, nor its variance was altered for mefloquine (MQ) and that the third column is included simply to make this point clear and to act as an internal ‘control’ demonstrating that neither the mean nor variance had changed for MQ.

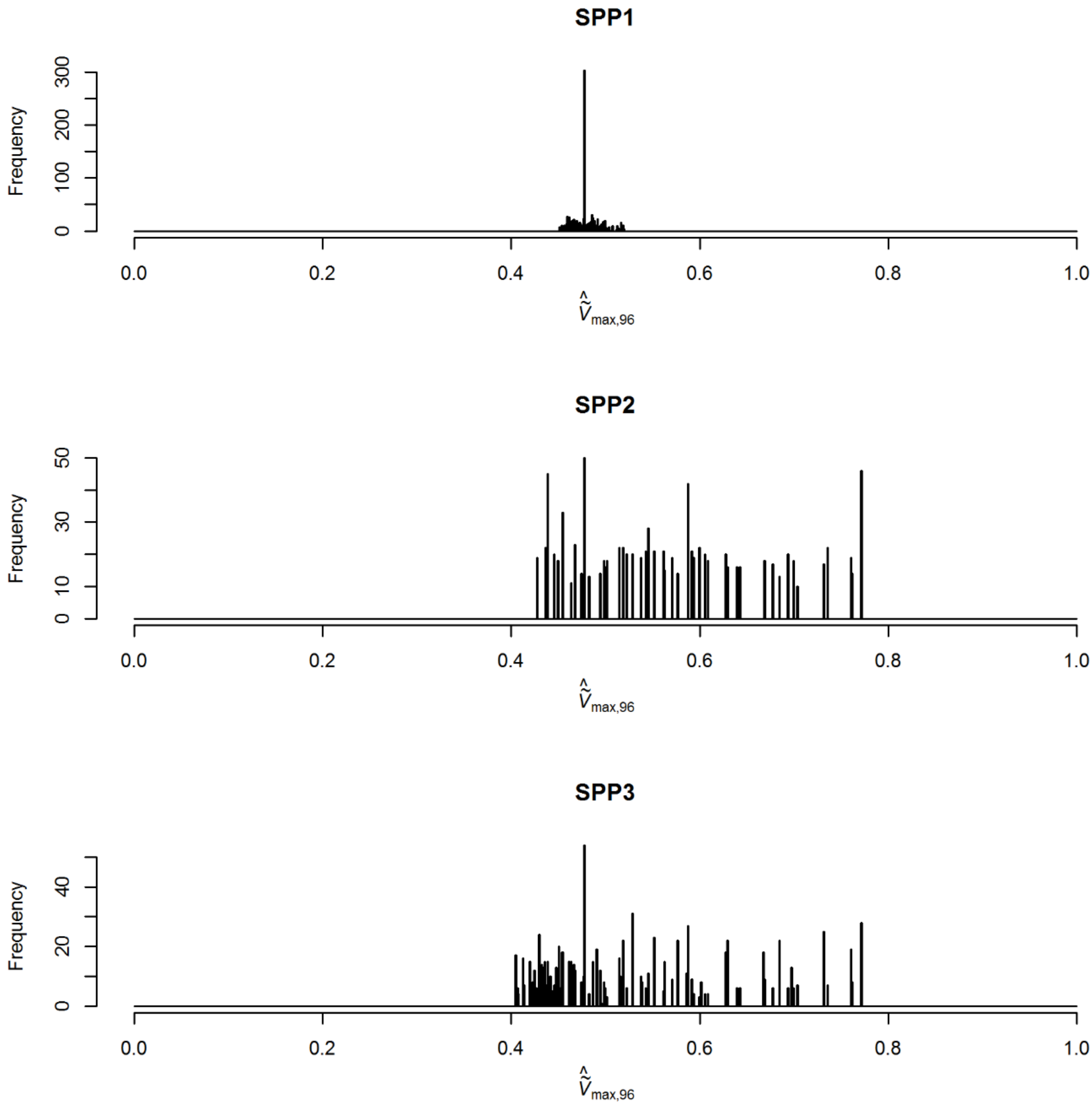

Figure S7. How patients' differing bin distribution at time of treatment introduces variance into the artemisinin killing rate (measured as $\hat{\tilde{V}}'_{max,96}$). Note that all patients harbour infections with the same sensitivity profile to artemisinins (i.e. calibrated to give $PRR_{48} = 10^4$ on a uniform age-bin distribution using the iso-sensitive pharmacodynamic profile on Figure 1; see text for details) so differing age-bin distributions at time of treatment is the sole source of variation in artemisinin kill rates. The effect is illustrated for three hypothetical parasite age-bin distributions in patient populations as described in the main text, i.e. for a simulated patient populations in a high transmission setting (SPP1), in a low transmission setting with good clinical infrastructure (SPP2) and in a low transmission setting with poor clinical infrastructure (SPP3).

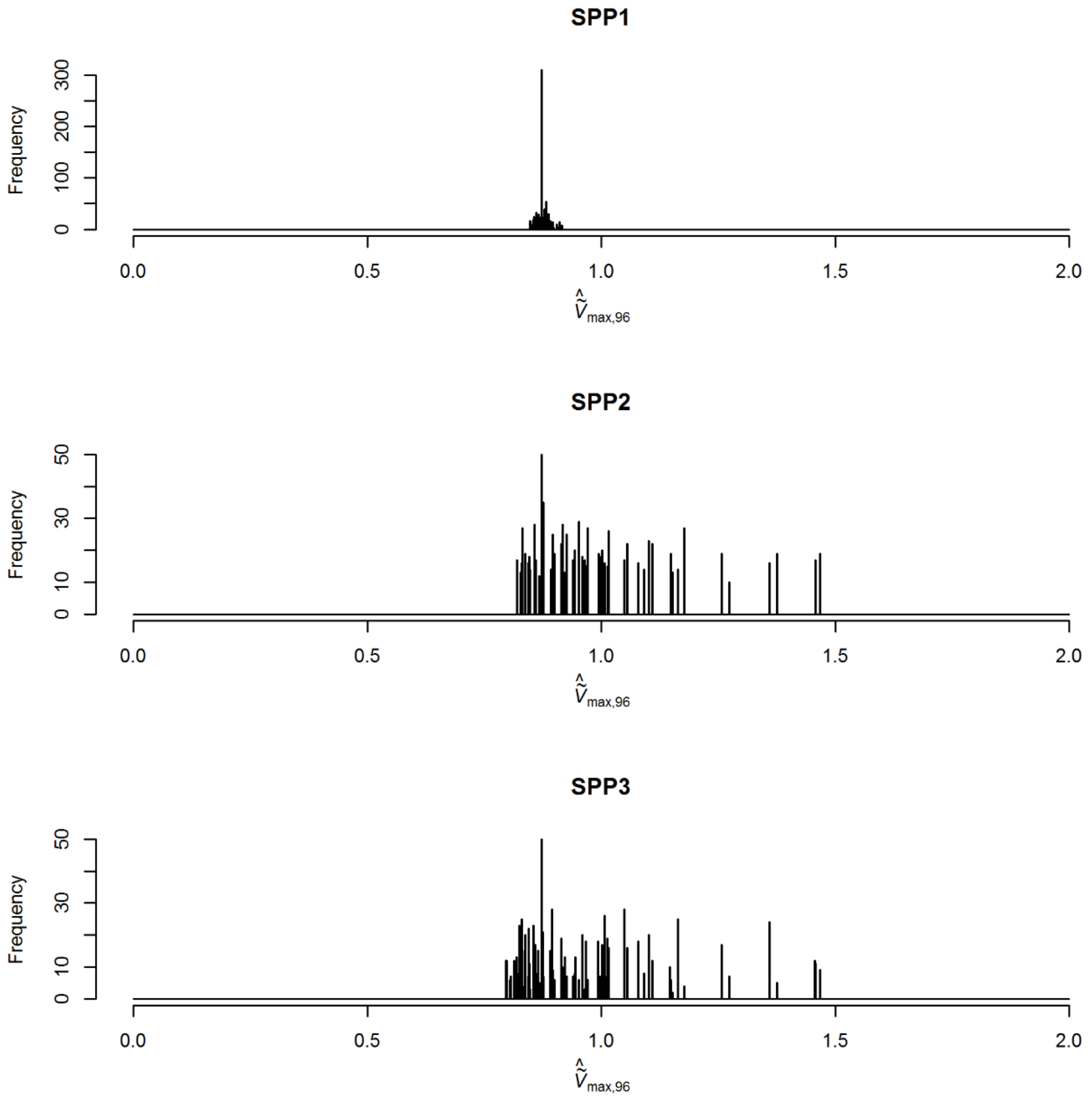

Figure S8. Variance in artemisinin killing with high mean kill rate ($\hat{\tilde{\mu}}_{\max}$). As for Figure S7 but with the value of the artemisinin killing rate, $\hat{\tilde{V}}'_{max}$, doubled so that $\hat{\tilde{V}}'_{max}$ = 2.33. The values of the equivalent continuous time killing rate, $\hat{\tilde{V}}'_{\max,96}$, along the x-axis illustrate the effects of parasites initial age-bin distribution on killing rate.

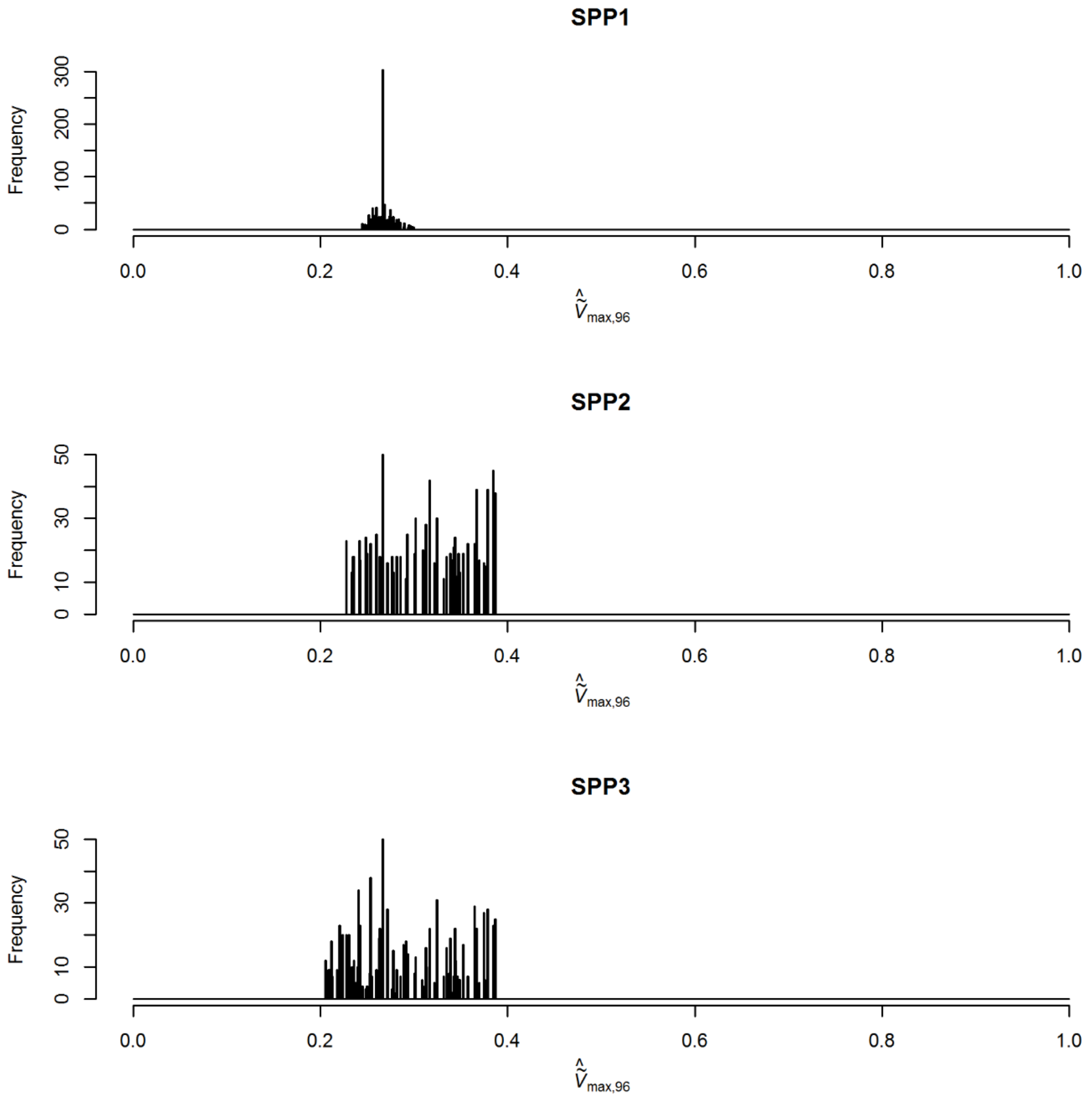

Figure S9. Variance in artemisinin killing with low mean kill rate ($\hat{\tilde{\mu}}_{\max}$). As for Figure S7 but with the value of the artemisinin killing rate, $\hat{\tilde{V}}'_{max}$, halved so that $\hat{\tilde{V}}'_{max}$ = 0.58. The values of the equivalent continuous time killing rate, $\hat{\tilde{V}}'_{\max,96}$, along the x-axis illustrate the effects of parasites initial age-bin distribution on killing rate.

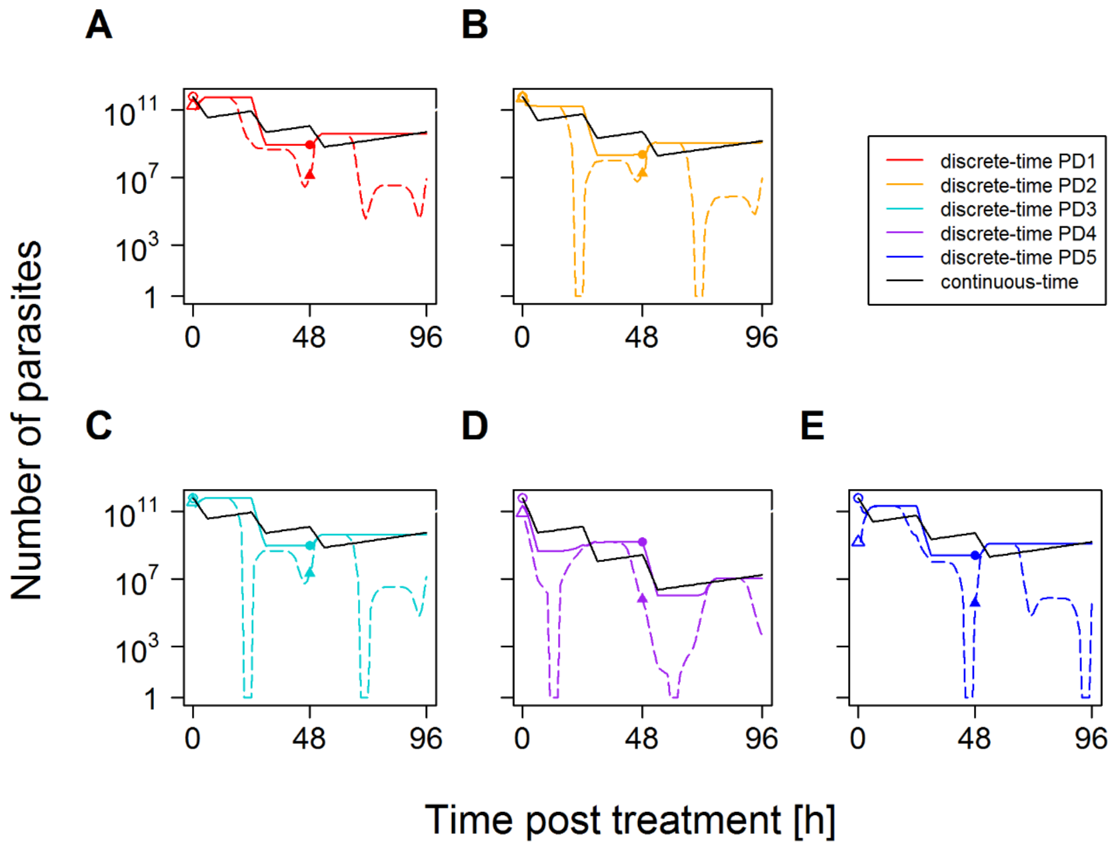

Figure S10. Total and circulating parasite numbers in paradigm distributions 1-5 following artemisinin treatment at times 0, 24 and 48 hours post-treatment. This shows total parasite numbers (solid lines) over time (as in Figure 4 of the main text) and the number of circulating (i.e. observable) parasite (dashed lines). The symbols indicate true (circles) or observable (triangles) parasite numbers at start of treatment (empty symbols) and at 48 hours (filled symbols) used to calculate the true and apparent parasite reduction ratio in Table 2 of the main text. Note that the true reduction in parasite numbers at 48 hours is always less than the reduction in observable, circulating parasites.

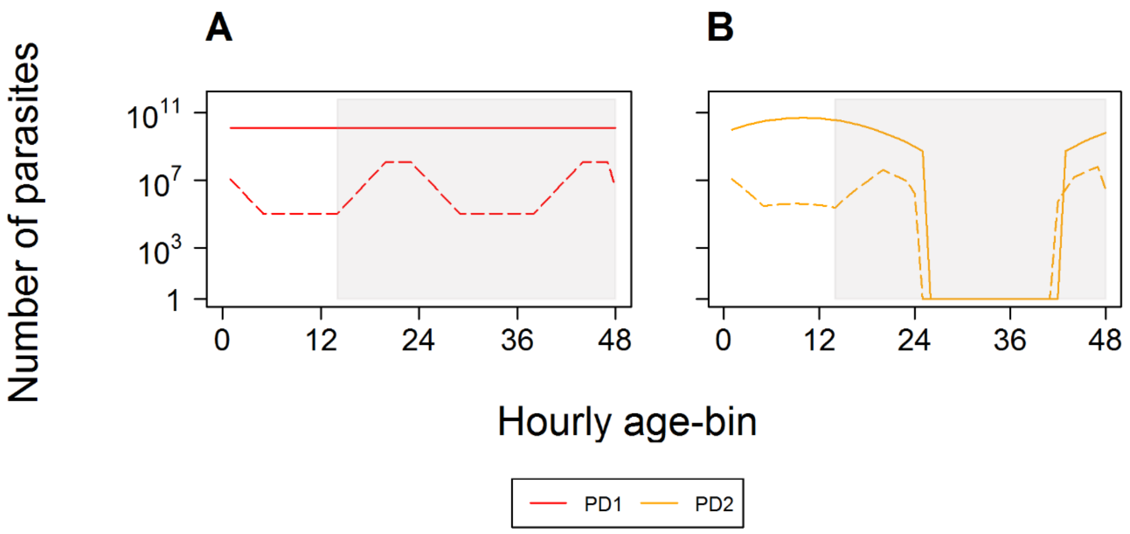

Figure S11. Effect of artemisinin killing on the age-bin distribution of parasites. This illustrates the number of parasites in each age-bin at the start of treatment and 48 hours later for two of the paradigm distributions, i.e. **(A)** PD1 and **(B)** PD2, with daily doses of an artemisinin i.e. at times 0 and 24 hours after treatment. The short pulses of stage specific artemisinin killing significantly alter the age-bin distribution between the start of treatment (solid lines) and the census period 48 hours later (dashed lines). Depending on the initial age-bin distribution of the parasites the magnitude of the effect on observable, circulating parsites (age-bins up to 14) and sequestered parasites (age-bins 15 and above, grey background) varies greatly. Note that the starting age-bin distribution in (B) has a large discontinuity; this arises because we limit the distribution to ± 15 hours around the mean and is largely an artefact of the log scale because at the point of discontinuity the number of parasites in the bins are already <10^{-3} those of the maximum value (the distribution is, of course, indistinguishable from normal on a arithmetic scale).
